# Rejuvenation of the aged brain immune cell landscape in mice through p16-positive senescent cell clearance

**DOI:** 10.1038/s41467-022-33226-8

**Published:** 2022-09-27

**Authors:** Xu Zhang, Vesselina M. Pearsall, Chase M. Carver, Elizabeth J. Atkinson, Benjamin D. S. Clarkson, Ethan M. Grund, Michelle Baez-Faria, Kevin D. Pavelko, Jennifer M. Kachergus, Thomas A. White, Renee K. Johnson, Courtney S. Malo, Alan M. Gonzalez-Suarez, Katayoun Ayasoufi, Kurt O. Johnson, Zachariah P. Tritz, Cori E. Fain, Roman H. Khadka, Mikolaj Ogrodnik, Diana Jurk, Yi Zhu, Tamara Tchkonia, Alexander Revzin, James L. Kirkland, Aaron J. Johnson, Charles L. Howe, E. Aubrey Thompson, Nathan K. LeBrasseur, Marissa J. Schafer

**Affiliations:** 1 Robert and Arlene Kogod Center on Aging, Mayo Clinic, Rochester, MN USA; 2 Department of Physical Medicine and Rehabilitation, Mayo Clinic, Rochester, MN USA; 3 Department of Physiology and Biomedical Engineering, Mayo Clinic, Rochester, MN USA; 4 Division of Clinical Trials and Biostatistics, Department of Quantitative Health Sciences, Mayo Clinic, Rochester, MN USA; 5 Department of Neurology, Mayo Clinic, Rochester, MN USA; 6 Department of Laboratory Medicine and Pathology, Mayo Clinic, Rochester, MN USA; 7 Mayo Graduate School and Medical Scientist Training Program, Mayo Clinic, Rochester, MN USA; 8 Department of Immunology, Mayo Clinic, Rochester, MN USA; 9 Department of Cancer Biology, Mayo Clinic Comprehensive Cancer Center, Mayo Clinic, Jacksonville, FL USA; 10 Ludwig Boltzmann Research Group Senescence and Healing of Wounds, Vienna, Austria; 11 Department of General Internal Medicine, Mayo Clinic, Rochester, MN USA; 12 Center for Multiple Sclerosis and Autoimmune Neurology, Mayo Clinic, Rochester, MN USA; 13 Division of Experimental Neurology, Mayo Clinic, Rochester, MN USA

**Keywords:** Microglia, Neural ageing, Neuroimmunology, Senescence

## Abstract

Cellular senescence is a plausible mediator of inflammation-related tissue dysfunction. In the aged brain, senescent cell identities and the mechanisms by which they exert adverse influence are unclear. Here we used high-dimensional molecular profiling, coupled with mechanistic experiments, to study the properties of senescent cells in the aged mouse brain. We show that senescence and inflammatory expression profiles increase with age and are brain region- and sex-specific. *p16*-positive myeloid cells exhibiting senescent and disease-associated activation signatures, including upregulation of chemoattractant factors, accumulate in the aged mouse brain. Senescent brain myeloid cells promote peripheral immune cell chemotaxis in vitro. Activated resident and infiltrating immune cells increase in the aged brain and are partially restored to youthful levels through p16-positive senescent cell clearance in female *p16-InkAttac* mice, which is associated with preservation of cognitive function. Our study reveals dynamic remodeling of the brain immune cell landscape in aging and suggests senescent cell targeting as a strategy to counter inflammatory changes and cognitive decline.

## Abstract (web summary)

The authors discovered that proinflammatory senescent myeloid cells may recruit peripheral immune cells in the aged mouse brain. Their findings implicate senescent cell clearance as a strategy to counter aged brain inflammation and cognitive decline.

## Introduction

Inflammation is a pervasive feature of aging with distinct innate and adaptive immune cell types playing instructive roles in tissue dysfunction. In the brain, microglia are multi-functional, resident immune cells, and throughout life, monocytes, macrophages, dendritic cells, neutrophils, B cells, and T cells, populate parenchyma and border regions^1^. Their function is to maintain homeostasis by responding to damage and stress; however, shifts in immune cell state and abundance may underlie age-dependent changes in brain health and function^2–4^.

In the aged and neurodegenerative brain, disease-associated microglia (DAM) and white matter-associated microglia (WAM) display features of exhaustion and activation, including downregulation of homeostatic genes (*Cx3cr1, P2ry12, P2ry13, Tmem119*) and upregulation of *Itgax/Cd11c*, major histocompatibility complex (MHC) components, neurodegenerative risk factors (*Apoe, B2m, Fth1, Lpl, Tyrobp*), chemokines and cytokines (*Ccl2, Ccl3, Ccl4, Spp1*), and lysosomal stress factors (lipofuscin [LF] granules, *Lgals3*)^1,3,5–10^. Despite a speculated role in mediating damage resolution, DAM/WAM may chemoattract peripheral immune cells, collectively resulting in inflammatory activation and maladaptive disruption of microenvironments in the aged brain. New strategies to deplete or rejuvenate DAM and WAM populations that may reinforce sterile brain inflammation could have important therapeutic potential.

Senescence is a cell fate assumed in response to stress and a conserved mechanism of tissue aging. It heterogeneously manifests in many cell types and contexts, characterized by macromolecular and organelle damage (including lysosomal changes, DNA damage, proteome instability), cell cycle arrest mediated by cyclin-dependent kinase inhibitors (including p16^Ink4a^, p21^Cip1^), activation of anti-apoptotic pathways (including BCL-2, serpines), and production of a senescence-associated secretory phenotype (SASP) (including chemoattractants, remodeling factors)^11–14^. The use of transgenic *p16-InkAttac* mice, which enable systemic elimination of p16-positive senescent cells, and senolytic drugs, which enable senescent cell killing by targeting anti-apoptotic pathways, have demonstrated that senescent cell clearance improves several domains of age-related tissue decline^15–19^, including central nervous system dysfunction^20–28^.

We and others hypothesized that a p16-related senescent mechanism may regulate age-related brain dysfunction. Using a tau-dependent neurodegeneration and *p16-InkAttac*-coupled model, Bussian et al. identified glial population positive for the lysosomal stress marker senescence-associated β-galactosidase (SA-β-gal), *p16*, and SASP factors and demonstrated that systemic targeting of p16-positive senescent cells reduces neuropathology and cognitive decline^22^. Also using *p16-InkAttac* mice, Ogrodnik et al. found that *p16*- and SASP-positive microglia accumulate in the aged brain and are associated with markers of brain inflammation^27^. Importantly, the molecular identity of p16-positive senescent microglia within the emerging brain immune landscape and the mechanistic role of systemic cell senescence in regulating steady-state brain inflammation have not been reported to our knowledge. Furthermore, although senescent cells and their SASP are accepted as drivers of age-related tissue inflammation^12,29^, whether and how their targeting may remodel immune cell composition in age-affected tissues, including the brain, is unknown.

Here, we leveraged single-cell mapping, region-specific transcriptional profiling, fluorescent imaging, and behavioral testing to study age- and sex-dependent steady-state brain cell senescence and inflammation in the context of late-life cognitive changes. Our findings reveal remarkable age-related diversity in brain inflammatory activation profiles at the cellular and transcriptional levels across the sexes. Through in vitro and in vivo experiments, we demonstrate that senescent brain myeloid cells are characterized by a SASP that drives peripheral immune cell chemotaxis. Importantly, we show that systemic targeting of p16-positive senescent cells partially rejuvenates brain immune cell composition, which is associated with the preservation of cognitive function.

## Results

### Senescence and inflammatory gene signatures are differentially upregulated in the aged mouse brain across sexes and brain regions

**Fig. 1:**
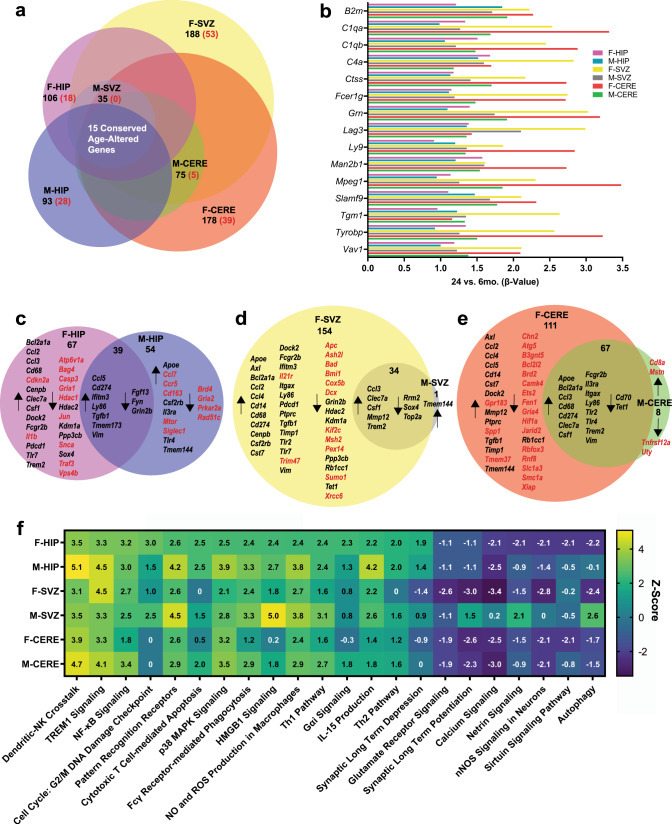
Gene expression signatures of senescence and inflammation are differentially regulated in the aged mouse brain across sexes and brain regions. **a** NanoString nCounter transcriptional profiling was used to assess age-, sex, and brain region-dependent differences in gene expression for inflammation- and senescence-related genes (*n* = 5 for young female and male groups, *n* = 6 for old female and male groups, with the same brains studied per hippocampus [HIP], subventricular zone-enriched striatum [SVZ], and cerebellum [CERE] sample). Black numbers indicate the total number of young *versus* old differentially expressed genes per region and sex. Red numbers indicate the number of genes that were distinctly expressed as a function of age in a single brain region and sex (linear regression model, false discovery rate (FDR) *q* < 0.05). **b** 15 genes were significantly different in young *versus* old comparisons across all brain regions and both sexes (*β*-value, indicative of the degree of change in outcome variable for every 1-unit of change in predictor variable; *q* < 0.05). Comparison of genes significantly up- or down-regulated as a function of age revealed transcripts that were commonly altered across more than one region or sex comparison (black) or distinctly altered in a single age, sex, and region comparison (red) in the **c** hippocampus, **d** subventricular zone, or **e** cerebellum. **f** Comparative Ingenuity pathway analysis (IPA) analysis of total probed inflammation- and senescence-related genes revealed *z*-scores for canonical pathways predicted to be up- (yellow) or down- (blue) regulated in the old *versus* young brain per region and sex. Source data are provided as a Source Data file.

To explore the influence of age on steady-state inflammation and senescence, we analyzed the expression of 800 genes using the NanoString nCounter mouse neuroinflammatory codeset and a custom senescence and aging codeset in the hippocampus, subventricular zone enriched-striatum, and cerebellum of 6-month-old (young) versus 24-month-old (old) female and male mice. Among females, we identified 274 age-influenced differentially expressed genes (*q* < 0.05), with 188 in the subventricular zone, 178 in the cerebellum, and 106 in the hippocampus (Fig. 1a). Among males, we identified 141 age-influenced genes, with 35 in the subventricular zone, 75 in the cerebellum, and 93 in the hippocampus (Fig. 1a). Fifteen conserved genes increased with age across regions and sexes (Fig. 1b), and 13 of 15 (*B2m*, *C1qa*, *C1qb*, *Ctss*, *Fcer1g*, *Grn*, *Lag3*, *Ly9*, *Man2b1*, *Mpeg1*, *Slamf9*, *Tyrobp*, and *Vav1*) were linked to brain myeloid cell activation, including DAM/WAM profiles^5,8,9,30–32^. Next, we studied the age- and region-specific expression patterns that were conserved and distinct between sexes and identified genes exclusively differentially expressed in a single brain region and sex (Fig.1a, c–e). Age-dependent expression profiles in the hippocampus appeared the most distinct between sexes (Fig. 1c). For the subventricular zone and cerebellum, males shared the majority of differentially expressed genes with females, with a greater proportion of female age-altered genes not identified in males (Fig. 1d, e).

Since distinct aging signatures defined each sex and brain region, we next considered all young versus old gene comparisons for ingenuity pathway analysis (IPA) (Fig. 1f). We observed consistent age-dependent increases in inflammatory pathways and decreases in synaptic-transmission pathways, with specific pathways distinctly regulated across brain regions and sexes. In the old female hippocampus, we observed pronounced activation of senescence-associated G2/M DNA damage checkpoint regulation (Fig. 1f), and related to this, *Cdkn2a/p16*^*Ink4a*^, a master regulator of the senescence program, was exclusively upregulated in the old female hippocampus at *q* < 0.05 (Fig. 1c). NF-kB signaling, which mediates the SASP, was also upregulated in the old female hippocampus. Autophagy and netrin signaling, pathways regulating cellular homeostasis and synaptic plasticity, respectively, were downregulated in the old female hippocampus. Dendritic and natural killer (NK) cell crosstalk and TREM1 signaling were robustly activated in the old male hippocampus. In the old female subventricular zone, TREM1 signaling was also strongly activated, and calcium signaling and synaptic long-term potentiation were downregulated. In the old male subventricular zone, HMGB1 signaling, and pattern recognition receptor function were upregulated. In the cerebellum, conserved pathways, including NF-kB signaling, dendritic and NK cell crosstalk, and p38 MAPK signaling, were upregulated (Fig. 1f).

Across sex- and region-specific aging expression profiles, we observed age-dependent upregulation of additional genes involved in brain myeloid cell activation and DAM/WAM states, including *Apoe, Cd274/Pd-l1, Fcgr2b, Ifitm3, Ly86, Ptprc/Cd45, and Trem2* (Fig. 1c–e). We also observed upregulation of genes associated with senescence and the SASP, many of which overlapped with myeloid cell activation, including *Axl, Bcl2a1a, Ccl2, Ccl3, Ccl4, Ccl5, Cdkn2a, Cenpb, Csf1, Il1b, Itgax, Mmp12, Spp1, Tgfb1, Tmem173/Sting*, and *Vim* (Fig. 1c–e). Upregulated senescence and SASP genes were enriched in the old female datasets (Fig. 1c–e). Downregulated expression profiles were distinct across datasets and included genes implicated in epigenetic regulation, mitochondrial function, apoptosis, and neurotransmission (Fig. 1c–f). Among key mediators of neurotransmission, we identified age-dependent downregulation of *Gria1* and *Grin2b*, particularly in the hippocampus, which is consistent with our prior findings^33^. We confirmed age-dependent reductions in GRIA1 and GRIN2B receptor abundance using immunofluorescence (IF) imaging and interestingly, observed hippocampal subregion-specific decreases in both old female and male mice (Supplementary Fig. 1). Thus, age-associated upregulation of senescent and inflammatory gene signatures distinctly characterize the aged brain across sexes and brain regions and coincides with the downregulation of key mediators of synaptic transmission.

### p16-positive senescent brain myeloid cells exhibit markers of altered chemoattractant SASP, MHC, lysosomal, and homeostatic function

**Fig. 2:**
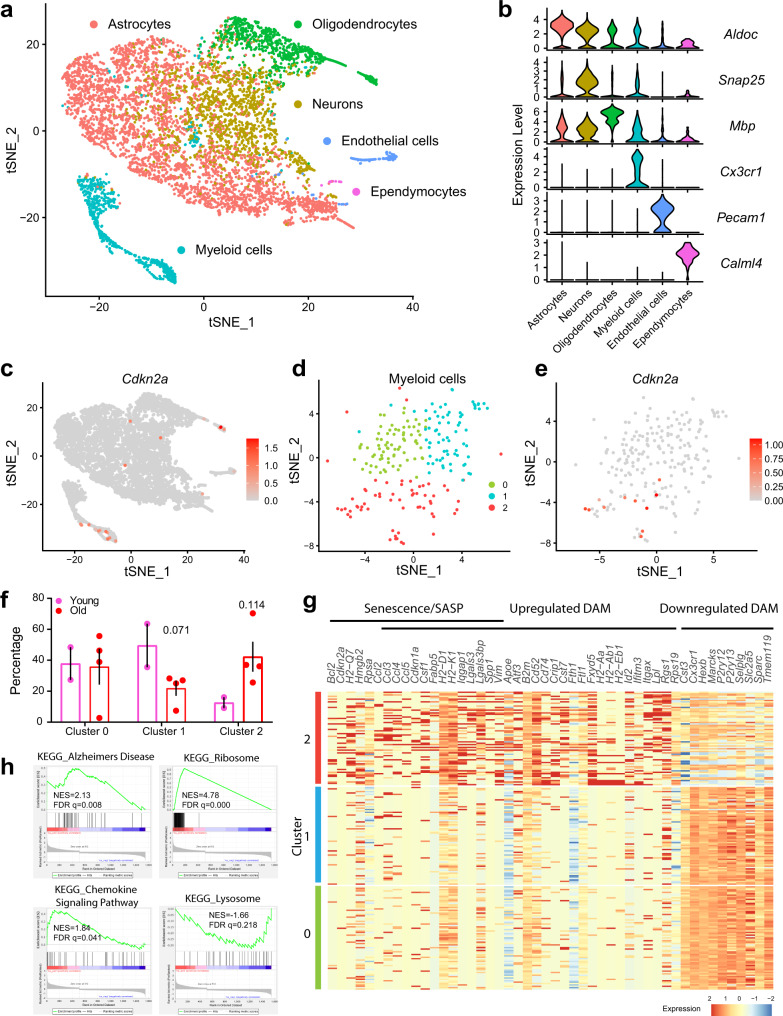
Single-cell RNA-sequencing reveals an age-related *Cdkn2a/p16*^*Ink4a*^-positive senescent myeloid brain cell population exhibiting a transcriptional profile of altered chemoattractant, MHC, lysosomal, and homeostatic function. **a** T-distributed stochastic neighbor embedding (tSNE) plot of cell types identified by scRNA-seq in mouse brain (*n* = 2 young and *n* = 4 old samples). **b** Violin plot of cell type marker expression within the six cell types. **c** tSNE plot showing *Cdkn2a/p16*^*Ink4a*^ expression in the six cell types. Each dot represents an individual cell. The color scale bar represents the *Cdkn2a* expression level. **d** tSNE plot of the three clusters of myeloid cells identified by sequential, unbiased subclustering with each cluster indicated by a distinct color. **e** Scatterplot depicting *Cdkn2a* expression in subclustered myeloid cells. Each dot represents an individual cell, and the clusters match panel (**d**). The color scale bar represents the *Cdkn2a* expression level. **f** Cell abundance of myeloid cells subclusters between 6- versus 24-month-old females (*n* = 2 for young, *n* = 4 for old; mean ± SEM; two-tailed unpaired *t*-tests). **g** Heatmap of expression levels of senescence, SASP, and disease-associated (DAM) genes in myeloid subclusters 0, 1, and 2. **h** Pathways enriched in cluster two identified by GSEA with the normalized enrichment score (NES) and FDR *q*-values indicated in each panel. Source data are provided as a Source Data file.

We next searched for senescent cell identities using single-cell RNA-sequencing (scRNA-seq) in young versus old mouse brains, focusing on females due to their more pronounced senescence-related transcriptional signatures (Fig. 1). Using the 10X droplet-based method, we recovered 6385 cells from two young and four old brain single-cell suspensions. Using the Seurat analysis pipeline, we identified six distinct cell clusters (Fig. 2a) defined by T-distributed stochastic neighbor embedding (tSNE) mapping of established transcriptional identities^34,35^, including astrocytes (*Aldoc*), neurons (*Snap25*), oligodendrocytes (*Mbp*), myeloid cells (*Cx3cr1*), endothelial cells (*Pecam1*), and ependymocytes (*Calml4*) (Fig. 2b, Supplementary Fig. 2a, b). An interactive website of the scRNA-seq data can be found at https://mayoxz.shinyapps.io/Brain/.

Based on our bulk gene expression observation that the senescence-effector *Cdkn2a/p16*^*Ink4a*^ was upregulated in the old female brain, we searched for the *Cdkn2a* transcript among defined populations. Myeloid cells contained the highest frequency of *Cdkn2a*-positive cells, with astrocytes, oligodendrocytes, and endothelial cell populations containing fewer *Cdkn2a*-positive cells (Fig. 2c). Given the pronounced abundance of *Cdkn2a* signal, we conducted unbiased sequential clustering of the myeloid compartment and identified a single-cell population that was enriched for *Cdkn2a*-positive cells, denoted ‘cluster two’ (Fig. 2d, e, Supplementary Fig. 2c–e), which trended to increase with age (Fig. 2f). Interestingly, *Cdkn2a*-positive cells in cluster two mapped with microglial (*Tmem119*) and monocyte (*Ly6c2*) markers (Supplementary Fig. 2c–e), suggesting that both resident and infiltrating cells contribute to a *Cdkn2a*-enriched brain immune population.

The transcriptional signature of cluster two was defined by the upregulation of both canonical senescence genes and overlapping gene signatures implicated in myeloid cell activation. This included upregulation of cyclin-dependent kinase inhibitors (*Cdkn2a, Cdkn1a*), senescence-related antiapoptotic factors (*Bcl2*), chemoattractant SASP factors (*Ccl2-5, Spp1*), MHC genes (*Cd74, H2-Aa, H2-Ab1, H2-Eb1, H2-D1, H2-K1, H2-Q7*), and lysosomal stress markers (*Lgals3, Lgals3bp*). Additional DAM/WAM genes were upregulated (*Apoe, B2m, Cst7, Fth1*, *Ifitm3, Itgax, Lpl*) and downregulated (*Cx3cr1, Hexb, Marcks, P2ry12, P2ry12, Selplg, Tmem119*) in cluster two (Fig. 2g). This signature also overlapped with age-related genes in the bulk regional NanoString transcriptional profiles (Fig. 1). Functional assessment based on gene set enrichment analysis (GSEA) revealed downregulation of lysosomal function and upregulation of chemokine signaling, ribosomal function, and Alzheimer’s disease (Fig. 2g, h).

To validate scRNA-seq results and specifically interrogate the contribution of microglia to the senescent myeloid cell compartment in an aged brain in a larger dataset, we analyzed scRNA-seq data previously published by Ximerakis et al. corresponding to eight young (2–3 months) and eight old (21–23 months) mouse brain single cell suspensions, which were processed through a pipeline very similar to our experiment^36^. Application of the Seurat analysis pipeline to 34,753 single brain cells revealed 14 distinct cell clusters defined by tSNE mapping of established transcriptional identities^34,35^ (Supplementary Fig. 3a). We identified a *Tmem119*-positive microglial population (Supplementary Fig. 3a, b), which we applied to unbiased subclustering (Supplementary Fig. 3c). Of the five subclusters, Ximerakis’s microglia cluster one contained the highest frequency of *Cdkn2a*-positive cells (Supplementary Fig. 3c, d). The abundance of Ximerakis’s microglia cluster one significantly increased in the aged brain (Supplementary Fig. 3e). Remarkably consistent with the gene expression profile of myeloid cluster 2 (Fig. 2g), Ximerakis’s microglia cluster one was characterized by expression of senescence-related cyclin-dependent kinase inhibitors (*Cdkn2a, Cdkn1a*), senescence-related antiapoptotic factors (*Bcl2*), chemoattractant SASP factors (*Ccl2-5, Spp1*), MHC genes (*Cd74, H2-Aa, H2-Ab1*), and lysosomal stress markers (*Lgals3*). Additional DAM/WAM genes were upregulated (*Cst7, Ifitm3, Itgax, Lpl*) and downregulated (*Cx3cr1, Hexb, P2ry12, P2ry13, Selplg, Tmem119*) (Supplementary Fig. 3f). GSEA revealed similar functional assessments to myeloid cluster two (Fig. 2h), with Ximerakis’s microglia cluster one characterized by downregulation of lysosomal function and upregulation of the senescence-related p53 pathway, chemokine signaling, cytokine-cytokine receptor interactions, and ribosomal function (Supplementary Fig. 3g). Cumulatively, two independent scRNA-seq datasets reveal a brain microglia cell population as a *Cdkn2a/p16*^*Ink4a*^-positive population in the old brain, characterized by overlapping gene signatures of senescence and DAM/WAM activation.

### p16-positive senescent myeloid cells accumulate in the aged, female dentate gyrus

We next explored the identity and abundance of p16-positive myeloid cells in the young and old brains of *p16-InkAttac* mice treated with vehicle (V) or AP20187 (AP) to systemically eliminate p16-positive cells^15^. We used RNA in situ hybridization (RNAish) to probe *Cdkn2a*/*p16*^*Ink4a*^ and IF imaging to probe IBA1, a marker of resident and infiltrating brain myeloid cells. Based on the transcriptional signature of lysosomal alterations identified in ‘cluster two’ cells by scRNA-seq, we also tracked LF granules as a marker of senescence-related lysosomal stress. We focused on the hippocampal dentate gyrus, an adult neurogenic niche susceptible to deleterious inflammatory remodeling in the aged brain^4^. In old female mice, we frequently identified *p16*^*Ink4a*^+IBA1+ cells, which trended to be 3.3-fold more abundant than in young females (Supplementary Fig. 4a, b). The majority of *p16*^*Ink4a*^+IBA1+ cells were also positive for LF granules (Supplementary Fig. 4a, c). Importantly, we discovered the necessity for assessing (or quenching) LF signal to accurately quantify *p16*^*Ink4a*^ punctae, due to signal overlap resulting from the broad autofluorescence inherent to LF granules (Supplementary Fig. 4a). Among females, no age- or treatment-dependent differences in IBA1-*p16*^*Ink4a*^+ cell abundance were identified (Supplementary Fig. 4d).

### Senescent brain myeloid cell SASP chemoattracts peripheral immune cells in vitro

**Fig. 3:**
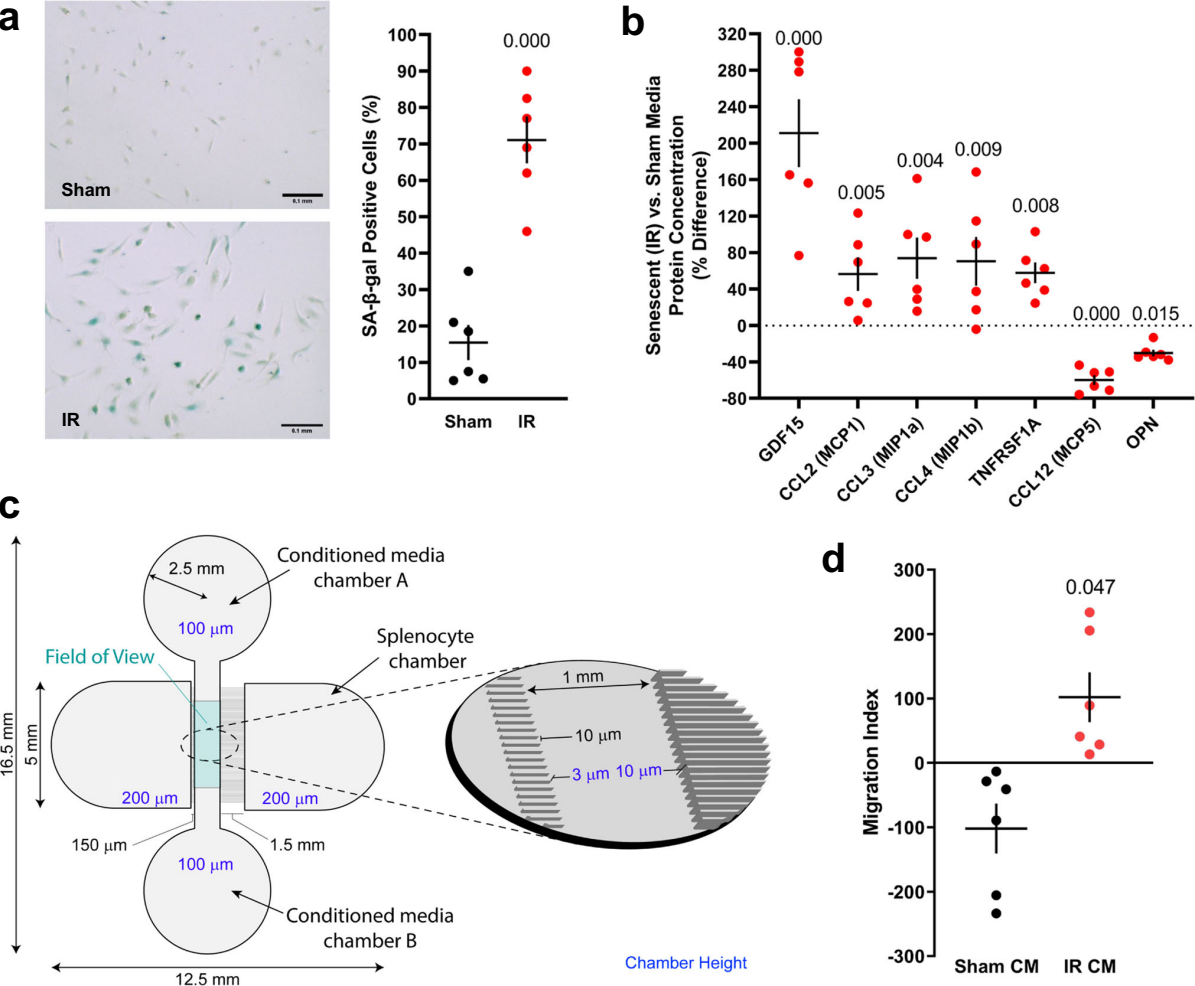
Senescent brain myeloid cells secrete a SASP that promotes peripheral immune cell recruitment in vitro. IR exposure to primary mouse brain myeloid cells induced senescence, which was confirmed by **a** SA-β-gal staining (scale bar: 0.1 mm) and **b** analysis of SASP proteins in conditioned media (mean ± SEM; two-tailed unpaired *t*-tests). **c** Schematic representation of the microfluidic migration chamber. The splenocyte entry chamber is connected via 10-micron grooves to the central imaging field. Conditioned medium from senescent or control brain myeloid cells was added to the indicated side chambers. **d** Migration index indicating average frame-to-frame migration toward SASP or control media from images collected every three min for 12 h (mean ± SEM; two-tailed one-sample *t*-test). See Supplementary Fig. 6 for a representative video of splenocyte migration to senescent cell media containing the SASP. (Data were generated from two separate experiments of *n* = 3.) Source data are provided as a Source Data file.

Based on our observation that *p16*-positive senescent myeloid cells in the aged brain upregulate chemoattractant SASP factors (Fig. 2), we devised an in vitro model to test whether the SASP of senescent brain myeloid cells drives peripheral immune cell chemotaxis. Exposure of primary mouse brain myeloid cells to 10 Gy X-ray irradiation (IR)-induced senescence over two weeks (Fig. 3a, b; Supplementary Fig. 5). Specifically, 71% of IR-exposed brain myeloid cells were positive for the lysosomal stress marker SA-β-gal (Fig. 3a). Among IR-exposed cultures, RT-PCR gene expression profiling revealed significant increases in the senescence effector *p16*^*Ink4a*^ and SASP factors *Ccl4 (Mip1b)* and *Mmp9*, as well as trends for increases in *Ccl3 (Mip1a)*, *Tnfa*, and MHC II-associated invariant chain *Cd74* (Supplementary Fig. 5). Analysis of secreted proteins revealed significant increases in established and/or chemoattractant SASP factors, including GDF15 (211% increase), CCL2/MCP1 (56% increase), CCL3/MIP1a (74% increase), and TNFRSF1A (58% increase) in the media of senescent IR-exposed cultures, relative to sham control media (Fig. 3b). Comparison of gene expression and secreted protein data revealed chemoattractant factor CCL4 was upregulated at both the gene expression and secreted protein levels, while GDF15 was robustly upregulated in the secreted protein pool but not transcriptionally, suggesting that the SASP is dynamically regulated.

Comparison of in vitro senescent brain myeloid cultures with scRNA-seq results (Fig. 2) demonstrated common upregulation of *p16*^*Ink4a*^, MHC II components, CCL2, CCL3, and CCL4, suggesting the in vitro model recapitulates critical chemoattractant features of in vivo senescent brain myeloid cluster two. We also noted model differences, including upregulation of *Spp1* in cluster two in vivo and downregulation of OPN (encoded by *Spp1*) in senescent myeloid culture media (Fig. 3b). We next assayed whether the senescent brain myeloid SASP chemoattracts peripheral immune cells using a microfluidic migration chamber system (Fig. 3c). GFP-positive splenocytes, consisting of T and B lymphocytes, dendritic cells, natural killer cells, neutrophils, and monocytes^37^, preferentially migrated toward senescent media containing SASP proteins (Fig. 3d, Supplementary Fig. 6 Video File). Thus, the SASP of senescent brain myeloid cells drives peripheral immune cell chemotaxis.

### Activated resident and infiltrating immune cells accumulate in the aged brain and are reduced by systemic targeting of p16-positive senescent cells

**Fig. 4:**
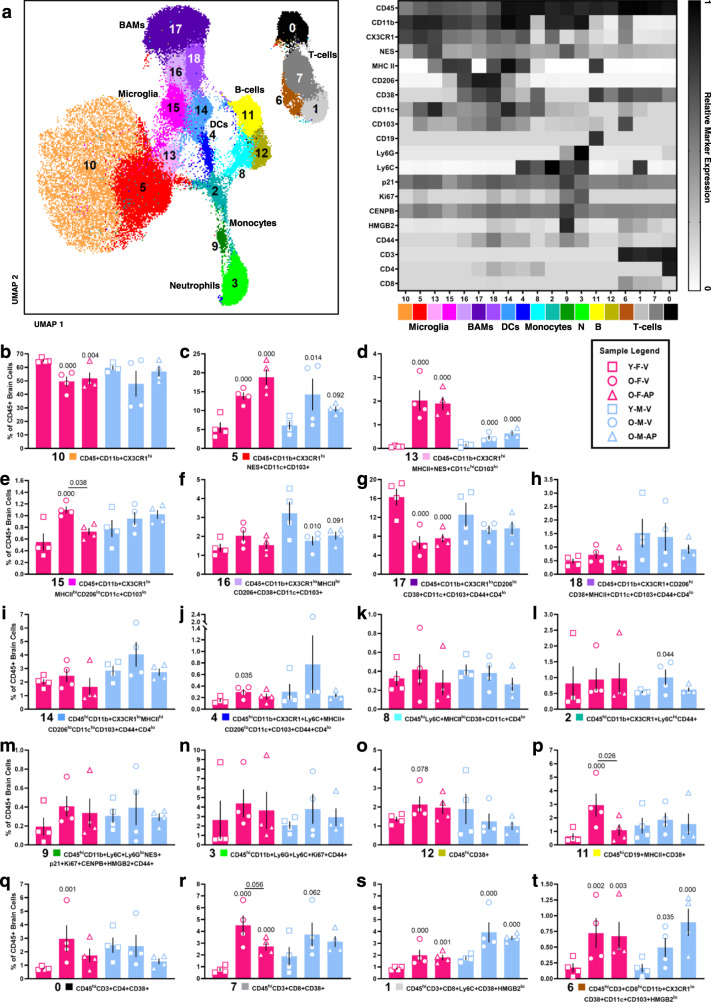
Mass cytometry demonstrates the increased frequency of activated resident and infiltrating immune cells in the aged brain, which are reduced by systemically targeting p16-positive senescent cells in females. **a** Uniform manifold approximation and projection (UMAP) representation of total CD45+ brain cell populations identified by mass cytometry with the colors and numbers corresponding to cell types delineated by FlowSOM-guided clustering, including distinct microglial, border macrophage (BAMs), B cell (B), dendritic cell (DCs), monocyte, neutrophil (N), and T cell populations defined by discrete marker combinations and proximity. **b**–**t** Frequency distributions of FlowSOM-delineated populations with numbers and color coding corresponding to panel (**a**) (*n* = 4; Y-F-V = 6 months, female, vehicle; O-F-V = 24 months, female, vehicle; O-F-AP = 24 months, female, AP20187; Y-M-V = 6 months, male, vehicle; O-M-V = 24 months, male, vehicle; O-M-AP = 24 months, male, AP20187; mean ± SEM; generalized linear mixed model with multiple comparison testing; female and male samples analyzed separately). Source data are provided as a Source Data file.

We next implemented high-dimensional mass cytometry to study age-related changes in brain immune cell composition and test whether systemic targeting of p16-positive senescent cells reduces the abundance of infiltrating immune cells and microglia exhibiting DAM features. Twenty immune markers were used to study changes in the frequency of cell types and states within the total CD45+ brain cell compartment in young *versus* old female and male *p16-InkAttac* mice treated with V or AP (Fig. 4; Supplementary Fig. 7). The total number of CD45+ cells identified in brain single-cell suspensions from young and old mice treated with V or AP did not differ in females or males (Supplementary Fig. 8). Uniform manifold approximation and projection (UMAP) dimensionality reduction and FlowSOM-guided clustering revealed remarkable immune cell diversity, including populations defined by both distinct and gradient marker expression, which differed by age, treatment, and sex. Seven broad cell type classifications were identified based on canonical marker abundance and cluster proximity, including microglia (CD45+CD11b+Ly6C−Ly6G-CD3−), border macrophages (BAMs: CD45+CD11b+CD206+CD38+), dendritic cells (DCs: CD45^hi^CD11c+CD103+), B cells (B: CD45^hi^CD19+), neutrophils (N: CD45^hi^Ly6C+Ly6G+), monocytes (CD45^hi^Ly6C+), and T cells (CD45^hi^CD3+). The seven clusters were further segregated based on the type and state markers for a total of 19 distinct populations (Fig. 4a).

Eleven CD45+ populations differed between old versus young female brains (Y-F-V versus O-F-V), and six CD45+ populations differed between old *versus* young male brains (Y-M-V *versus* O-M-V). Two populations that increased in frequency in old females were significantly reduced following AP treatment (O-F-V versus O-F-AP). In old females, we observed a significant decrease in the frequency of CD45+CD11b+CX3CR1^hi^ ‘homeostatic’ microglia, which was the most abundant CD45+ population (population 10; Fig. 4b). The age-related reduction in homeostatic microglia was accompanied by an expansion of activated resident microglia and infiltrating immune cells. Among both males and females, we observed age-dependent increases in frequency of two activated microglial populations, which were CD45+CD11b+CX3CR1^hi^NES+CD11c+CD103+ (population 5; Fig. 4c) and CD45+CD11b+CX3CR1^hi^MHCII+NES+CD11c^hi^CD103^lo^ (population 13; Fig. 4d). An additional microglial population increased in frequency in aged females, which was CD45 + CD11b + CX3CR1^lo^MHCII^hi^CD206^lo^CD11c+CD103^lo^ (population 15; Fig. 4e). Microglia population 15 exhibited a marker expression pattern overlapping with scRNA-seq senescent myeloid cluster two (Fig. 2g) and Ximerakis’s cluster 1 (Supplementary Fig. 3f), characterized by DAM/WAM signature downregulation of CX3CR1 and upregulation of MHCII and CD11c (encoded by *Itgax*). Importantly, the abundance of population 15 was reduced in aged females treated with AP. Thus, systemic senescent cell targeting reduces the abundance of a microglial population exhibiting an overlapping molecular signature of senescence and DAM/WAM activation.

We identified three putative BAM populations co-expressing CD206 and CD38 (populations 16–18; Fig. 4f–h). Of the BAM clusters, a CD45+CD11b+CX3CR1^lo^MHCII^hi^CD206+CD38+CD11c+CD103+ population decreased in frequency in the aged male brain (population 16; Fig. 4f), and a CD45+CD11b+CX3CR1^lo^CD206^hi^CD38+CD11c+CD103+CD44+CD4^lo^ population decreased in frequency in the aged female brain (population 17; Fig. 4g), which may reflect sex differences in the age-dependent activation state of BAMs. CD45^hi^CD11b+CX3CR1^lo^MHCII^hi^CD206^lo^CD11c^hi^CD103+CD44+CD4^lo^ DCs did not statistically differ with age in either sex (population 14; Fig. 4i). A CD45^hi^CD11b+CX3CR1+Ly6C+MHCII+CD206^lo^CD11c+CD103+CD44+CD4^lo^ putative plasmacytoid DC population increased in frequency in the aged female brain (population 4; Fig. 4j). Three Ly6C+ monocyte populations (populations 8, 2, 9; Fig. 4k–m) and one Ly6G+Ly6C+ neutrophil population (population 3; Fig. 4n) were identified, which displayed variability in females. CD45^hi^CD11b+CX3CR1+Ly6C^hi^CD44+ brain monocytes increased in aged males treated with V (population 2; Fig. 4l). CENPB and p21 are markers that have been previously used to identify senescent cells and here, identified a distinct CD45^hi^CD11b+Ly6C+Ly6G^lo^NES+p21+Ki67+CENPB+HMGB2+CD44+ monocyte population (population 9; Fig. 4m). A CD45^hi^CD38+ population was also identified; since the population was CD45^hi^, these cells are likely other infiltrating immune cells that were not clearly delineated with the markers included on our panel (population 12; Fig. 4o). CD45^hi^CD19+MHCII+CD38+ B cells increased in frequency in the aged female brain, and AP treatment reduced their abundance, demonstrating an infiltrating immune population that was restored to youthful levels in the aged female brain following senescent cell clearance (population 11; Fig. 4p).

Four CD45^hi^CD3+ T cell populations were identified, all of which were positive for CD38. All T cell populations increased in the aged female brain, and two increased in the aged male brain (populations 0, 7, 1, 6; Fig. 4q–t). CD45^hi^CD3+CD4+CD38+ cell abundance increased in the brains of aged females treated with V (population 0; Fig. 4q). The age-dependent increase in CD45^hi^CD3+CD8+CD38+ cells trended towards a reduction in females treated with AP (population 7; Fig. 4r). CD45^hi^CD3+CD8+Ly6C+CD38+HMGB2^lo^ cells increased in frequency in the aged brain in both sexes (population 1; Fig. 4s). The least abundant T cell population identified was CD45^hi^CD3+CD8^hi^CD11b+CX3CR1^lo^CD38+CD11c+CD103+HMGB2^lo^. Despite the implementation of an analysis pipeline to eliminate doublets or aggregates, coexpression of canonical T cell markers with other myeloid markers may indicate that this population was comprised of interacting cells, but interestingly, this population increased in frequency in both the aged female and male brain (population 6; Fig. 4t).

Our mass cytometry results demonstrate dynamic remodeling of the inflammatory cell landscape in the old *versus* young brain, with more pronounced expansion of activated resident microglia and infiltrating immune cell compartments in aged females, relative to males. AP treatment to systemically target p16-positive senescent cells significantly reduced the abundance of a microglia population coexpressing MHCII and CD11c (population 15; Fig. 4e), which demonstrated remarkable marker similarity to the senescent myeloid cluster two and Ximerakis’s microglia cluster one identified by scRNA-seq (Fig. 2g, Supplementary Fig. 3f). Transgenic senescent cell targeting also attenuated age-dependent increases in infiltrating immune populations, including B cells and T cells.

### Cognitive function is preserved following p16-positive senescent cell targeting and associated with abundance of activated microglia and infiltrating brain immune populations in aged female mice

**Fig. 5:**
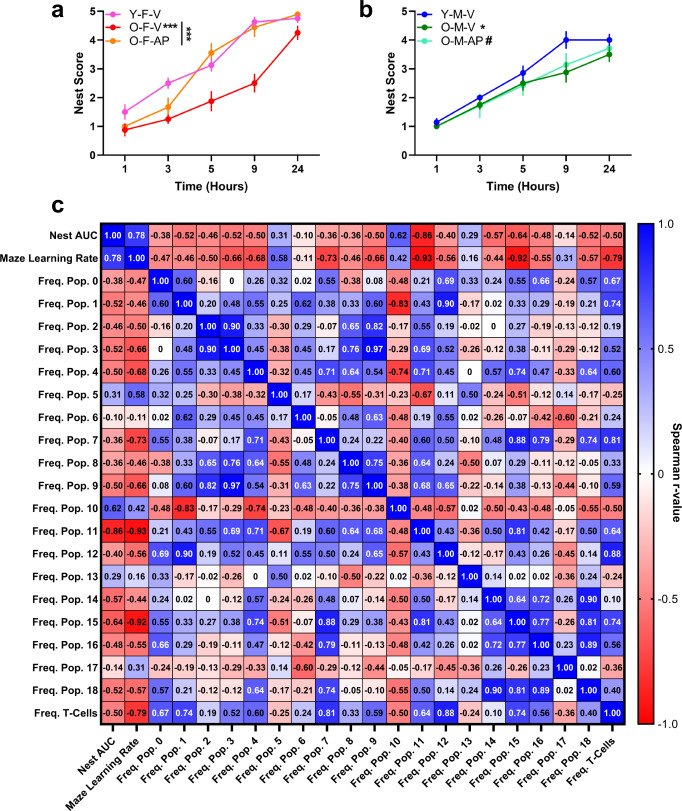
Cognitive function is preserved by p16-positive senescent cell targeting and associated with the abundance of activated resident microglia and infiltrating brain immune populations in aged females. **a** Nest building was assessed as a measure of executive function, using an established quality scale of 0-5 over 24 h among six- (Y) *versus* 24- (O) month-old **a** females (F) or **b** males (M) treated with vehicle (V) or AP20187 (AP) (*n* = 8 Y-F-V, *n* = 8 O-F-V, *n* = 9 O-F-AP, *n* = 7 Y-M-V, *n* = 8 O-M-V, *n* = 7 O-M-AP; mean ± SEM; two-way ANOVA; ***p = 0.000, *p = 0.013, ^#^*p* = 0.052). **c** Spearman correlation *r*-values defining associations between nest score area under the curve (AUC), stone maze learning rate [(trial 1 errors−trail 2 errors)/trial 1 errors], CD45+ brain immune populations defined in Fig. 4, and total CD3+ T cell frequency. Source data are provided as a Source Data file.

Nest building is translationally equivalent to an ‘activity of daily living’ and an indicator of hippocampal-integrated executive cognitive function that is impaired in 2-year-old mice^38^. We found that both old female and male mice built significantly poorer quality nests more slowly than young mice, with a more pronounced age difference in females (Fig. 5a, b). Nest building quality and speed in AP-treated old females did not differ relative to young females and were significantly better than in V-treated old females, suggesting a marked improvement in female executive function in response to systemic senescent cell targeting (Fig. 5a). As a secondary cognitive outcome, we implemented the water-motivated stone maze task to probe hippocampal-related decision making, learning, and memory^39^. Similar to nest building, we identified a performance benefit in AP-treated old females, relative to V-treated old females, but not males (Supplementary Fig. 9a, b). AP-treated old females made fewer navigational errors than V-treated old females, which was most apparent in the first two trials (Supplementary Fig. 9a). No treatment-dependent differences in stone maze navigational speed were identified for either sex (Supplementary Fig. 9c, d).

We next implemented Spearman correlation analyses to test whether the abundance of activated brain immune cell populations was associated with cognitive outcomes. We focused analyses on aged females treated with AP or V to specifically interrogate the influence of senescent cell elimination on interactions between brain immune cell composition and cognitive changes (Fig. 5c). Nest building performance (nest score area under the curve (AUC)) was robustly and inversely correlated with the abundance of B cell population 11 (*r* = −0.86, *p* = 0.011). We also observed a trend to an inverse correlation between the frequency of the putative senescent microglia population 15 and nest AUC (*r* = −0.64, *p* = 0.096). We calculated a learning rate variable from the stone maze data by subtracting trial 2 errors from trial 1 errors and dividing by trial 1 errors [(T1−T2)/T1] and found that the stone maze learning rate was associated with nest AUC (*r* = 0.78, *p* = 0.029), suggesting that executive function and spatial learning and memory may be distinct but linked cognitive domains that can be improved by senescent cell elimination. Stone maze learning rate was negatively associated with the abundance of B cell population 11 (*r* = −0.93, *p* = 0.002) and microglia population 15 (*r* = −0.92, *p* = 0.003) and interestingly, was also negatively associated with the abundance of total brain T cells (sum of populations 0, 7, 1, 6; *r* = −0.79, *p* = 0.025) and T cell population 7 (*r* = −0.73, *p* = 0.048) (Fig. 5c). Thus, activated resident and infiltrating brain immune cell abundance is strongly related to cognitive dysfunction in aged females.

The abundance of the putative senescent microglia population 15 positively correlated with the abundance of B cell population 11 (*r* = 0.81, *p* = 0.022), T cell population 7 (*r* = 0.88, *p* = 0.022), and total T cells (*r* = 0.74, *p* = 0.046), among other activated infiltrating immune populations (Fig. 5c). This is consistent with the notion that senescent brain microglia may recruit peripheral immune cells through a chemoattractant SASP, which collectively contributes to age-related inflammatory circuitry disruption driving the cognitive decline. Importantly, this cellular inflammatory imbalance was partially restored by systemically targeting p16-positive senescent cells (Fig. 4).

## Discussion

Our study sheds light on dynamic shifts in the age- and sex-dependent immune and senescent brain cell landscape in step with cognitive changes. Importantly, we demonstrate that systemically targeting p16-positive senescent cells through a transgenic approach is a strategy to attenuate age-related brain inflammatory remodeling linked to cognitive decline.

Using NanoString transcriptomic profiling of inflammatory and senescence gene panels, we discovered that females and males display distinct and shared age-dependent expression signatures across brain regions, with a more divergent hippocampal pattern. The total number of age-altered genes was greater in females, which may reflect a combination of an amplified aging response, lower baseline expression in young females, and/or less expression variability, relative to males. We identified a conserved signature of DAM/WAM-related myeloid cell activation across aged brain regions and sexes. At the pathway and gene levels, we observed age-related activation of senescence-related gene signatures, particularly in the aged female hippocampus. Activation of senescence and DAM/WAM signatures coincided with reductions in glutamatergic receptor mRNA and protein abundance.

Using scRNA-seq, we identified an age-associated myeloid population denoted ‘cluster two’ that was characterized by an expression signature of senescence, chemoattractive SASP, lysosomal stress, and MHC activation, which overlapped with previously reported DAM/WAM profiles^1,3,5–10^. Pathway analyses revealed alterations in cytokine, lysosomal, and ribosomal function, as well as a predicted link to Alzheimer’s disease. We corroborated scRNA-seq findings using an independent dataset generated from young and old mice of similar ages and experimental approaches^36^. Comparative analysis using this larger dataset enabled a clearer focus on the microglia-specific brain myeloid compartment and validated our observation that a senescent microglia population accumulates in the aged mouse brain. Taken together, our regional and single-cell transcriptomics analyses suggest senescent myeloid cells accumulate in the aged brain and may be an important source of chemoattractant factors driving age-related brain inflammation. Using fluorescent imaging, we confirmed the presence of *p16*^*Ink4a*^-positive myeloid cells in the female dentate gyrus hilus, which trended to be more abundant in aged mice. *p16*^*Ink4a*^-positive myeloid cells harbored LF granules, which may also be associated with increased Galectin 3 (encoded by *Lgals3*, upregulated in scRNA-seq myeloid cluster two and Ximerakis’s microglia cluster one), a chemoattraction-related factor that binds lysosomal β-galactoside and β-galactosidase^40–42^, a classical cell senescence marker^43^, collectively implicating lysosomal stress as a feature and biomarker of a senescent myeloid state. Using an in vitro system, we demonstrated that IR-induced senescent brain myeloid cells recapitulate features of senescent brain myeloid cells in vivo, including harboring lysosomal stress markers and upregulating and secreting chemoattractant SASP factors. Importantly, we showed that splenocytes migrated toward SASP-containing conditioned media from senescent brain myeloid cells, but not control cell media. Steady-state mouse splenocytes are primarily comprised of T cells (~40+%) and B cells (~20+%)^37^, which suggests these cell types may be recruited by the senescent brain myeloid cell SASP. The specific peripheral immune cell identities and SASP molecules mediating their chemotaxis are important future directions.

Based on the observations that p16-positive senescent brain myeloid cells accumulate in the aged brain in vivo and recruit peripheral immune cells in vitro, we implemented mass cytometry to study steady-state brain immune cell composition in young and aged female and male mice and importantly, interrogate the influence of systemic p16-positive senescent cell targeting. This revealed dynamic age-dependent remodeling of brain immune cell composition, which was partially prevented by senescent cell targeting, particularly in females. We observed a significant reduction in the abundance of non-activated or ‘homeostatic’ microglia in the aged brain, which coincided with the expansion of activated resident microglia and infiltrating inflammatory cell populations. We discovered a microglial population (cluster 15) characterized by DAM/WAM signature downregulation of CX3CR1 and upregulation of MHCII and CD11c that resembled the marker expression pattern of senescent myeloid scRNA-seq cluster two and Ximerakis’s cluster one. The abundance of this putative senescent brain microglial population increased in old females and was reduced to youthful levels in aged females treated with AP. Similarly, age-dependent expansion of T cells and B cells was attenuated or completely prevented, respectively, by senescent cell targeting in aged females. Critically, brain infiltrating T cells and B cells have been previously implicated as mediators of inflammation-related cognitive dysfunction and neurodegenerative disease through mechanisms that may involve direct interactions with DAM^44,45^, further substantiating our proposed mechanism and potential for its modulation through senescent cell targeting. Of note, the brain T cell and B cell populations identified through mass cytometry were positive for CD38, a master regulator of age-related metabolic dysfunction^46^, suggesting an alternative therapeutic avenue for modulating inflammatory cell composition in the aged brain. Through cognitive behavioral testing, we show that senescent cell targeting in aged females improved performance in executive function-linked nest building and learning and memory-linked stone maze navigation, relative to aged controls. Performance in these tasks was robustly associated with the abundance of senescent/DAM microglia and infiltrating B cells and T cells, ultimately demonstrating that age-related inflammatory brain cell composition is associated with cognitive dysfunction and can be partially restored through senescent cell clearance. These findings are foundational to our future investigations of the specific mechanisms by which senescent and infiltrating immune cells disrupt neuronal circuitry to drive cognitive dysfunction.

Our results suggest several scenarios by which senescent cell targeting may beneficially influence brain immune cell composition. First, p16-positive brain myeloid cells (resident microglia and infiltrating myeloid cells) may drive peripheral immune cell recruitment, and their targeting may prevent a shift to homeostatic imbalance, characterized by infiltration of activated, potentially senescent, circulating inflammatory cells. Second, other senescent cell types (e.g., p16-positive astrocytes, oligodendrocytes, and endothelial cells) may also exert proinflammatory influence and may also be cleared. Third, the systemic *p16-InkAttac* transgenic strategy may reduce the abundance of circulating senescent immune cells, thereby depleting the pool available for recruitment into the brain under steady-state inflammatory conditions. Fourth, systemic senescent cell elimination may reduce the abundance of senescent cells throughout the body that contributes SASP factors to the circulating progeronic proteome^47,48^, which is a driver of age-related brain dysfunction^49^. Based on the established influence of circulating senescent inflammatory cells and SASP factors as mediators of organ homeostasis and the systemic influence of the *p16-InkAttac* transgenic strategy^15,29,47^, we assert that rejuvenation of the inflammatory brain cell landscape and associated improvements in cognitive function following transgenic clearance of p16-positive cells in aged mice may reflect a combination of senescent cell elimination in the brain, periphery, and circulation. Importantly, we have studies underway designed to disentangle the mechanistic influence of systemic versus brain cell senescence. Finally, transgenic targeting of p16-positive cells may also eliminate cells that are p16-positive but not functionally senescent per se^50^. Through the use of cell-specific molecular profiling methods, we demonstrated that a predominant p16-positive brain cell type exhibits cell-intrinsic features of an exhausted state, including downregulation of homeostatic factors and upregulation of stress-related and canonical senescence-related factors. Continued adoption of cell-specific profiling in interventional studies will be essential for understanding the identities, targeting efficiencies, and mechanisms by which specific senescent cell populations contribute to organismal aging.

Our findings are consistent with and shed mechanistic light on other recent studies exploring the influence of senescent cells on brain dysfunction. Simultaneous to our experiments, Ogrodnik et al. showed exclusively in males over an older and wider age range of 27–31 months that systemic senescent cell clearance reduced the abundance of senescent brain myeloid cells, coincident with improvements in spatial-related decision-making^27^. Contrastingly, our aged experimental groups were 24 months old and at the onset of cognitive decline, wherein relative to males, females exhibited more pronounced age-related decline across transcriptional, cytometry, and behavioral outcomes, which were attenuated by transgenic senescent cell clearance. This suggests that distinct changes in age-related brain senescence-related inflammatory remodeling may present earlier in females, but importantly, senescent cell targeting may improve late-life cognitive function in both sexes, albeit with different temporal kinetics. Similarly, prior reports have demonstrated the accumulation of senescence markers in Alzheimer’s neurodegenerative models^21,22,51^, and other studies have demonstrated that activated immune cells may fail to clear and ultimately exacerbate proteopathy through cytokine signaling^45,52^, suggesting a mechanistic link to our findings. Exploring brain inflammatory dynamics at older ages and in neurodegeneration models while assessing the comparative responsivity of systemic and emerging cell-specific transgenic senescent cell targeting strategies, in addition to senolytic drugs with translational potential, are important for future studies. Furthermore, our scRNA-seq and mass cytometry data were generated using single-cell suspensions, and we acknowledge that fragile and/or interconnected cells, such as neurons, may have been inadvertently depleted in those analyses. To overcome this limitation, we are developing high parameter spatial imaging, which will reveal the unique molecular signatures of senescent cells within defined brain microenvironments in solid-state brain tissue throughout aging.

Sex differences in brain aging are an important area of investigation to which our results contribute new insights. Both our transcriptional and cytometry data revealed more robust senescence and inflammatory differences in females; however, we caution against over-interpreting this as a broad outcome. To accommodate a study design in which non-adolescent mice could be treated with a vehicle for several months, young mice were 6 months old at the endpoint, which may represent a middle-age timepoint corresponding to the onset of age-related changes. Furthermore, we qualitatively observed greater signal variability in aged males, relative to aged females, which could also contribute to fewer age-related differences identified in males. In future studies, it will be informative to map senescent cell identities and related inflammatory changes sequentially across the lifespan in females and males, which will shed light on the emergence of senescent cells, associated inflammatory states, and their conservation versus heterogeneity throughout the lifespan across sexes. A deeper investigation of the influence of sex hormones on senescent and inflammatory mechanisms is also warranted.

Collectively, our study reveals important overlap in emerging frameworks for mechanistically understanding brain aging at the cellular, molecular, and functional levels. We build on recent single-cell advances and demonstrate that steady-state inflammation in the aged brain is characterized by expansion of resident senescent DAM/WAM and infiltrating immune populations. Critically, we show that p16-positive senescent myeloid cells and their SASP may mediate peripheral immune cell recruitment to exacerbate brain inflammation in aging, and systemic elimination of p16-positive senescent cells may rejuvenate brain immune cell composition to a more youthful state while improving cognitive function. Thus, our findings implicate senescent cell targeting as a tractable strategy to combat age-related brain inflammation and preserve cognitive function.

## Methods

### Mouse models

Mouse experiments were performed under protocols approved by Mayo Clinic Institutional Animal Care and Use Committee (protocol #A00003875-18). Mice were cared for in compliance with the guidelines in the National Institutes of Health Guide for Care and Use of Laboratory Animals. Wild-type and *p16-InkAttac* mice (both C57BL/6 background) were used for in vivo studies. Mice were group-housed in ventilated cages with a constant temperature of 25 °C, 30–70% humidity, a 12-h light/dark cycle, and provided standard chow. As indicated in the results, we took advantage of the ability to systemically eliminate p16-positive cells through the administration of AP20187 (AP) to induce Caspase 8-mediated cell death^15^. For treatment studies, 18-month-old *p16-InkAttac* mice were randomized (based on body weight and body composition measured by echo-MRI) to receive V or AP (2 mg/kg) with a treatment strategy of 3 consecutive daily intraperitoneal injections per week, every other week (six treatments per month), until 24 months of age. Three-month-old *p16-InkAttac* young control mice received the same V dosing strategy from 3 to 6 months of age. Thus, all aged mice were treated with V or AP for six months, and young controls were treated with V for 3 months. We recognize a shorter V treatment strategy in young controls is a technical limitation, but we opted to prioritize initiation of treatment when young mice were developmentally mature and endpoint in young mice at 6 months of age since we view this as an early middle age timepoint. All mice were behaviorally phenotyped by investigators blinded to experimental groups in the weeks prior to necropsy, and treatments were discontinued at least one week prior to necropsy. At the time of necropsy, all mice were examined for gross pathology and tumor prevalence, with the exclusion of mice harboring pronounced splenomegaly. For in vitro chemotactic assays described below, splenocytes were harvested from 1 to 2 months old C57BL/6-Tg(CAG-EGFP)131Osb/LeySopJ mice (JAX stock #006567).

### Tissue collection and processing

Mice were euthanized with a lethal dose of pentobarbital and were transcardially perfused with ice-cold PBS. Brains were immediately removed and processed for downstream applications. Hemisected brain hemispheres allocated to NanoString nCounter analyses were immediately dissected under a microscope; the hippocampus, subventricular zone-enriched striatum, and cerebellum were stored at −80 °C. Brain hemispheres allocated to RNA in situ hybridization and IF imaging were drop-fixed in 4% paraformaldehyde for 24 h and then subjected to a sucrose gradient (24 h in 12% sucrose-PBS, 24 h in 18% sucrose-PBS, 72 h in 30% sucrose-PBS), followed by storage in cryopreservation media (30% glycerol, 30% ethylene glycol in PBS) at −80 °C. Brains were sectioned at 16 µm using a microtome prior to staining. For scRNA-seq and mass cytometry, single-cell suspensions were generated based on our optimization of a recently published method^36^. The cerebellum was removed, and the remaining brain hemisphere was rinsed with HBSS containing 1% glutamax, minced with a razor blade into ~10 pieces, and centrifuged in HBSS without calcium and magnesium-containing 1% glutamax and 5% trehalose at 220×*g* (2 min, room temperature). The supernatant was aspirated, enzyme mix 1 from the Adult Brain Dissociation Kit (Miltenyi Biotec) was added at half concentration, and the tube was incubated for 15 min at 34 °C at 150 rpm rotation. Enzyme mix 2 from the Adult Brain Dissociation Kit was added and the solution was dissociated by gentle titration with a 5 ml pipette, followed by 10 min at 34 °C under slow continuous rotation; this step was repeated one time. 10% ovomucoid protease inhibitor (Worthington-biochem, Lakewood, NJ) was added next, followed by 10 ml of wash buffer (HBSS with 0.5% BSA and 1% glutamax). This solution was filtered through a pre-wet 70 µm strainer with additional wash buffer added to flush cells through the filter. The filtered solution was passed through a pre-wet 40 µm strainer with additional wash buffer added to flush cells through and centrifuged at 220×*g* (8 min, room temperature), and the supernatant was aspirated. To deplete cell debris in the absence of high-speed centrifugation, the pellet was resuspended in 200 ml wash buffer and centrifuged for 220×*g* (8 min, room temperature). After aspirating the supernatant, the pellet was resuspended in FACS buffer (HBSS with 0.5% BSA, 1% glutamax, and 5% trehalose) and centrifuged for 220×*g* (8 min, room temperature). The final pellet was resuspended in PBS on ice and immediately processed for scRNA-seq or mass cytometry.

### NanoString nCounter gene expression

Regional brain microdissections were subjected to Trizol-based RNA extraction, followed by nanodrop concentration and purity analysis. RNA samples were applied to NanoString nCounter (NanoString Technologies, Seattle, WA) digital RNA quantification, using the predesigned mouse neuroinflammatory codeset and a spike in senescence and aging custom codeset, according to the manufacturer’s specifications. Analyzed genes are listed in Supplementary Dataset 1. Briefly, 100 ng of total RNA was hybridized to capture-reporter probe codesets and immobilized on NanoString cartridges. Excess RNA and probes were removed, and digital barcodes were counted. NanoString nCounter analysis software (v4.0) was used for counting, quality control, and normalization. Transcript counts were normalized according to NanoString’s recommendations. This approach utilized 12 housekeeping genes (*Aars, Ccdc127, Cnot10, Csnk2a2, Fam104a, Gusb, Lars, Mto1, Supt7l, Tada2b, Tbp, Xpnpep1*) and six positive control genes, representing a standard curve of expression abundance.

### Single-cell RNA-sequencing (scRNA-seq) and data analysis

Single-cell suspensions were counted using the Vi-Cell XR Cell Viability Analyzer (Beckman-Coulter, Brea, CA, USA) and ~5000 cells were loaded into the 10X Genomics Chromium system to generate Gel Beads-In-Emulsion (GEMs). Reverse transcription was performed using Chromium Single Cell 3’ Reagent Kits (v2, 10X Genomics, Pleasanton, CA, USA). Standard Illumina sequencing primers and a unique i7 Sample index were added to each cDNA for library construction. Libraries were sequenced using the Illumina cBot and HiSeq 3000/4000 PE Cluster Kit. The flow cells were sequenced as 100 × 2 paired-end reads on an Illumina HiSeq 4000 using HiSeq 3000/4000 sequencing kit and HCS v3.3.52 collection software. Base-calling was performed using Illumina’s RTA version 2.7.3. Cell Ranger (v3.0) (10X Genomics) was used for alignment to the mouse genome version mm10. R (v3.6.0) and Seurat (v3.1.3)^35^ were used for further analysis following the standard workflow. For quality control and filtering, cells with detected genes fewer than 200 or >8000, or with mitochondrial gene content >50% were excluded. For Ximerakis’s scRNA-seq dataset, R (v4.0.3) and Seurat (v4.0.2) were used. Gene set enrichment analysis was performed using software GSEA (v4.0.3)^53^. The interactive website for scRNA-seq data was generated using ShinyCell (v2.1.0)^54^.

### RNA in situ hybridization and immunofluorescence imaging

RNAish was performed following the RNAScope Multiplex Fluorescent Reagent Kit v2 protocol (323100-USM) (Advanced Cell Diagnostics, ACD). Fixed, frozen tissues were dehydrated in an alcohol cascade (50%, 70%, and two separate 100% Ethanol washes) for 5 min each. RNAscope Hydrogen Peroxide was applied to tissues and incubated at room temperature for 10 min, followed by treatment with RNAScope 1X Target Retrieval Reagent and incubated for 5 min at 95 °C. Tissues were then washed in 100% ethanol and dried at room temperature before creating a barrier. RNAScope Protease III was applied and incubated for 30 min at 40 °C in the HybEZ Oven, washed with water, and the target probe was prepared (*Cdkn2a/p16*;^*Ink4a*^ #447491-C2). Target probe was applied and incubated at 40 °C for 2 h, after which tissues were washed with 1X wash buffer and placed in 5X SSC buffer overnight. Tissues were washed with wash buffer twice followed by incubation with AMP1 (30 min 40 °C), AMP2 (30 min 40 °C), and AMP3 (15 min 40 °C). Tissues were washed twice in between each amplification step. After the amplification cascade, tissues were incubated with HRP specific to channel 1 probe (15 min 40 °C). Tissues were washed twice, after which the TSA fluorophore specific to the channel 1 probe was attached and incubated (30 min 40 °C). Tissues were washed twice and incubated with RNAScope HRP Blocker (15 min 40 °C). The HRP, TSA fluorophore, and HRP blocker steps were repeated with reagents and/or fluorophores specific to the channel 2 probe (*Cdkn2a/p16*^*Ink4a*^). Immunofluorescent staining was applied to the same tissues immediately following RNAish according to the ACD recommended protocol (323100-TN). Tissues were washed twice with wash buffer and twice with TBST wash buffer before being incubated with 10% Normal Goat Serum in TBS-0.1% BSA for 1 h at room temperature. Blocking reagents were removed and IBA1 primary antibody (019-19741; Wako, 1:100) or isotype control in TBS with 0.1% BSA was added and incubated at 4 °C overnight. Tissues were washed twice in TBST wash buffer, and Alexa Fluor 488 goat anti-rabbit IgG (H + L) (A11008; ThermoFisher/Invitrogen; 1:150) in TBS with 0.1% BSA was applied and incubated for 1 h at room temperature. Tissues were washed twice and DAPI was applied for 30 s at room temperature before mounting with Prolong Gold antifade and storing in the dark at 4 °C. Fluorescent images were captured using a Zeiss Axio Observer D1 microscope (Carl Zeiss, Oberkochen, Germany) with a ×20 objective, an Axiocam 702 camera, and FIJI software for quantification^55^. A threshold for each fluorescent channel was established based on both a negative and positive control slide. Post image collection, the upper and lower threshold values on the image histogram were adjusted to fit the line of best fit so that signal detection was optimal for quantification. Cells that were positive for *Cdkn2a/p16*^*Ink4a*^, IBA1, and/or autofluorescent LF granules (imaged at 568 nm) were quantified.

Immunofluorescent staining of AMPA glutamate receptor 1 (α-GRIA1/GluA1 rabbit monoclonal; Abcam, Catalog# ab183797, 1:1000) and NMDA glutamate receptor 2B (α-GRIN2B mouse monoclonal; Abcam, Catalog# ab93610, 1:500) was performed on fixed, frozen brain sections. First, sections were washed in blocking solution (8% donkey serum/0.1% Triton-X / 0.1% Tween-20 in PBS) for 2 h at room temperature. After two washes with PBS, TrueBlack® Plus LF autofluorescence quencher (Biotium, Catalog# 23014, 1X) was applied for 10 min, followed by three washes with PBS. Brain sections were then stained overnight with α-GRIA1 and α-GRIN2B antibodies at 4 °C. After washing three times with PBS, donkey α-mouse Alexa Fluor 488 (Jackson Immunoresearch, Catalog# 715-546-151, 1:250) and donkey α-rabbit rhodamine red-X (Jackson Immunoresearch, Catalog# 711-296-15, 1:250) F(ab’)2 fragment antibodies were applied to tissue for 2 h at room temperature. Sections were then washed three times with PBS, and Vectashield containing DAPI was then applied. Images were acquired with microscope and imaging software as above. Fluorescence intensity of each hippocampal sub-region was determined by corrected total fluorescence by background subtraction for each image (integrated density−(area*mean background)). Six to eight images were quantified and averaged per sub-region of each animal with 3 mice per group.

### Primary brain myeloid cell culture: isolation, purification, and senescence induction and confirmation

Mixed glial cells were isolated from P1 to P3 C57BL/6 mouse brains (adapted from ref. 56). Briefly, 2.5 brains were pooled together per culture. Cultures were incubated in DMEM medium supplemented with 10% fetal bovine serum (FBS) and 1% Penicillin–streptomycin for 4 days at 37 °C. Subsequently, mixed glial cultures were enriched for microglia cells in DMEM containing 10% FBS, 1% Penicillin–streptomycin, and 20% LADMAC (ATCC CRL-2420) conditioned medium (CM) for 14 days at 37 °C with media replenished every 2 days. After enrichment, the microglial cells were purified using magnetic-associated cell sorting (MACS) with magnetic beads (CD45+, CD11b+) with MS columns according to the Miltenyi Biotec protocols. Cells were counted using an automated cell counter before sorting and after each sort to evaluate cell count and viability with trypan blue, at a ratio of 1:1 with the cell suspension. Cells were seeded into 12-well plates at a density of 3 × 10^4^ cells/well for SA-β-gal staining and the remaining cells were seeded into T-75 flasks. All purified microglial cells were seeded into plates or flasks that were pre-coated with poly-d-lysine (25 μg/ml, Sigma P1024).

Senescence was induced at ~50% confluency by exposure to 10 Gy IR using an RS2000 X-ray machine with an automated duration. IR-exposed cells were cultured for two weeks before analysis, while shams were cultured until they reached a density of 55–60% before analysis. IR-exposed and sham cells were maintained in a DMEM medium containing 10% FBS, 1% Penicillin-streptomycin, and 20% LADMAC CM until the endpoint with media replaced every three days. For a collection of CM, shams and IR cells were switched to serum-free DMEM for 24 h. CM was collected and filtered using a 0.22 µm filter and aliquoted into 1.5 ml microfuge tubes. The abundance of SASP proteins in CM was analyzed using commercially available multiplex magnetic bead immunoassays (R&D Systems) based on Luminex xMAP multianalyte profiling platform and analyzed on a MAGPIX System (Merck Millipore), as previously described^47,57^. CM was applied to chemotactic migration assays, as described below. After CM collection, cells were rinsed in PBS and incubated with 1 ml of 0.25% trypsin at 37 °C to allow cell detachment. Trypsin was neutralized by adding 10 ml DMEM with FBS. Cells were counted using an automated cell counter and then centrifuged at 450×*g* for 5 min to yield a cell pellet, which was resuspended in 1 ml Trizol for RNA isolation.

In addition to analysis of the SASP in CM, SA-β-gal staining and RT-PCR were utilized for secondary and tertiary confirmation of senescence. SA-β-gal staining was conducted as previously described^47^. Briefly, cells were rinsed in PBS once and fixed for 10 min in 4% paraformaldehyde in PBS at room temperature. Cells were rinsed twice with PBS and incubated for 6 h in SA-β-gal staining solution (1 mg/ml X-gal, 40 mM citric acid/sodium phosphate buffer pH 6.0, 5 mM potassium ferrocyanide, 5 mM potassium ferricyanide, 150 mM sodium chloride, and 2 mM magnesium chloride in water) at 37 °C in the dark on a shaker. Cells were rinsed twice in PBS, stained with Hoechst dye in PBS for 2 min, and stored at 4 °C until imaging. Images were taken using bright field and DAPI channels on an EVOS microscope (3–5 images per well) with a ×10 objective. Percentages of SA-β-gal positive cells were calculated by taking the ratio of SA-β-gal positive cells relative to Hoechst positive using ImageJ software. RNA was isolated from microglia cell pellets using Trizol-based chloroform–isopropanol precipitation, followed by nanodrop concentration and purity analysis, and cDNA synthesis through M-MLV reverse transcription (Invitrogen, cat# 18091200). RT-PCR was performed on a Quantstudio5 RT-PCR system (ThermoFisher) with Taqman probes from Integrated DNA Technologies (IDT) (Supplementary Dataset 2). Experimental genes were normalized to *Hprt* housekeeping gene expression and average abundance of the sham condition.

### Microfluidic migration chamber assay and splenocyte preparation

Microfluidic chamber (MFC) dimensions are provided in Fig. 3c. Master molds were fabricated by photolithographic patterning of photoresist (SU-8) on silicon wafers. MFCs were replicated in polydimethylsiloxane elastomer (PDMS; Sylgard 184 Elastomer Kit, Dow Corning) by pouring a solution of one part curing reagent per 10 parts PDMS onto a master mold and incubating at 55 °C overnight. Cured PDMS slabs with imprinted fluidic architecture were cut from a master mold. Media reservoirs were created using a 4 mm biopsy punch (Miltex) on a cutting mat (Electron Microscopy Sciences). To sterilize, MFCs were autoclaved in glass Petri dishes and stored until use. Three MFCs were adhered to cover-glass bottom TC treated six-well plates (Cellvis; P06-1.5H-N) using a template for alignment with the IncuCyte camera. To remove air bubbles from interchamber grooves, 100% ethanol was flushed through all chambers. Ethanol was gradually replaced with ddH_2_O by iteratively transferring 30 µl to the proximal chamber, distal chamber, and left side port for the central chamber and removing fluid from the right port of the central chamber once the volume had equilibrated. In the same fashion, ddH_2_0 was subsequently replaced with complete media for lymphocytes (CML) consisting of RPMI 1640 (Invitrogen), 3% v/v heat-inactivated fetal bovine serum, 1% v/v Na Pyruvate, 1% v/v non-essential amino acids, 1% v/v Penicillin and Streptomycin, and 25 µM beta-mercaptoethanol. The absence of bubbles was confirmed visually and ddH_2_O was added between wells to limit evaporative loss before proceeding.

Splenocytes were prepared by mechanical dissociation of C57BL/6-Tg(CAG-EGFP)131Osb/LeySopJ mouse spleen in 37 °C CML media with a sterile 7 ml Dounce homogenizer (20 strokes). Cells were centrifuged at 400×*g* for 4 min at room temperature, followed by red blood cell (RBC) lysis 1 ml ACK (150 mM NH_4_CL, 10 mM KHCO_3_, 0.1 mM Na EDTA, pH 7.4) for 90 s at room temperature. Lysis was terminated by adding 9 ml HBSS, and cells were centrifuged for 3 min at 400×*g* and passed through a 40 µm cell strainer (Falcon; #352340) to obtain a single-cell suspension. Splenocytes were counted, centrifuged at 400×*g* for 4 min, and resuspended in CML at a final concentration of 2 × 10^8^ cells per ml. Prior to the addition of cells or conditioned media, CML was allowed to equilibrate across all chambers, and equilibrium was thereafter maintained by removing 20 µl media for a given chamber just prior to adding 20 µl cells or conditioned media. GFP + splenocytes (4 × 10^6^ in 20 µl) were added to the proximal chamber of the MFC. Immediately following, 20 µl of conditioned media from senescent (IR) or non-senescent (Sham) microglial cultures were added to the lateral compartments in an identical manner. Finally, 10 µl of additional CML media were added on top of the proximal chamber containing cells to confer a brief fluidic drive to push cells from the proximal chamber into the central chamber and six-well plates were immediately transferred to IncuCyte (Sartorius) for live cell imaging. A representative migration video is provided in Supplementary Fig. 6 Video File. Micrographs (×4 objective) were acquired every 3 min for 12 h using IncuCyte SX5 G/O/NIR Optical Module (300 ms exposure). Images were cropped to include only the central chamber and exported tiff series and were batch imported into time series in Imaris 9.8 (Oxford Instruments) for cell tracking analysis. After background subtraction and alignment, automated spot detection was performed based on pixel area, intensity, and quality score. Inbuilt autoregressive motion algorithms were used to identify cell migration tracks based on the following criteria: gap size <2, interval distance <3 cell lengths. Tracks with displacement <3 cell lengths (e.g., non-motile cells) were excluded. Our experimental design utilized paired comparison of migration toward senescent versus non-senescent conditioned media with randomization of media into chamber A versus B. Migration index was calculated as average cell displacement (in pixels) between frames (3 min) over the 12 h recording. Migration toward a given stimulus is indicated by positive values, whereas toward the alternative stimulus is indicated by negative values.

### Mass cytometry and data analysis

Mass cytometry (*aka* cytometry by time of flight or CyTOF) was used to detect 28 intracellular or extracellular markers (Supplementary Dataset 3). The Maxpar panel designer tool (Fluidigm Sciences Inc., USA) was used to assign metal tags to each antibody in the panel. Antibodies for each marker were purchased directly from Fluidigm or were conjugated with metal tags using the Maxpar labeling kit and were subsequently tested for specificity. Antibodies were titrated to minimize signal overlap into neighboring channels and cell type-specific markers were separated within the panel in a manner that minimized signal interference due to the known predictable overlap from isotopic impurities. Cell pellets were suspended in Maxpar cell staining buffer (CSB) (Fluidigm) and labeled with 0.5 μM cisplatin solution (Fluidigm). After cisplatin labeling, cells were centrifuged at 500×*g* for 5 min and washed twice with CSB. Cells were resuspended in an antibody cocktail including 19 cell surface antibodies and then incubated with gentle agitation at room temperature for 45 min. Afterward, cells were washed with CSB twice prior to fixation with 2% paraformaldehyde at room temperature with gentle agitation. After fixation, CSB was added, and samples were centrifuged at 800×*g* for 5 min at room temperature. After removal of the supernatant, cells were resuspended in PERM-S buffer (Fluidigm) and centrifuged at 800×*g* for 5 min. Cells were then resuspended in a PERM-S antibody cocktail containing nine intracellular antibodies and incubated at 4 °C with gentle agitation. Then cells were washed with CSB and fixed in 2% paraformaldehyde for 30 min with gentle agitation. After washing with CSB, cells were incubated with 62.5 nM solution of DNA intercalator (Cell-ID Intercalator; Fluidigm) diluted in Maxpar Fix/Perm Buffer (Fluidigm) and incubated at 4 °C overnight. On the following day, cells were washed with PBS prior to resuspension in a 1:10 mixture of EQ four-element calibration beads and Cell Acquisition Solution (Fluidigm). Cells were resuspended at a concentration of 500,000 cells per ml. Prior to analysis cells were strained through a 40 µm cell strainer to eliminate excess debris and cell aggregates. Labeled cell suspensions were introduced into the Helios mass cytometer at a rate no greater than 350 events per second to minimize cell doublets. Introduced cells were then nebulized and subjected to an inductively coupled plasma source to ionize the metal labeled cells which were then introduced into a time-of-flight mass spectrometry chamber. Signals corresponding to each marker were captured, integrated, and normalized to spiked-in EQ beads using CyTOF software (v7.0.5189).

Live cells were isolated and exported for downstream analyses by manually gating on beads, residual, center, offset, width, event length, live/dead (195Pt), DNA1 (191Ir), and DNA2 (193Ir)^58^. Parameters were hyperbolic arcsine (arcsinh) transformed and scaled. FlowSOM clustering^59^ utilizing 20 cell type markers, including 12 immune cell identity positive selection markers (CD45, CD11b, CD11c, CD103, CD38, CD206, CD19, Ly6C, Ly6G, CD3, CD4, CD8) and 8 negative cell identity selection markers (NEUN, SYP, CD171, SOX2, MPB, MOG, CD31, GFAP), was used to identify and isolate the total CD45+ compartment and exclude aggregates. Following, scales were rechecked and the total CD45+ compartment was dimensionally reduced by uniform manifold approximation and projection (UMAP)^60^ and FlowSOM clustered using 20 markers, which included both type/identity and state markers (CD45, CD11b, CD11c, CD103, CD38, CD206, CD19, Ly6C, Ly6G, CD3, CD4, CD8, CD44, CENPB, HMGB2, p21, CX3CR1, Ki67, MHC II [I-A/I-E], NES). See Supplementary Dataset 3 for label, scale, and identity information for each marker used in mass cytometry analyses. FlowJo (v10.8.1) with R (v4.1.2) was used for mass cytometry analyses.

### Nest building

Nest building was assessed as a measure of executive function, as previously described^61,62^. Mice were transferred to the testing room for acclimation at least 30 min before the experiment. One cotton nestlet (Ancare) was placed on a clean cage floor prior to placing the mouse in the cage, which contained sanichips free of any additional nesting material and ad libitum access to water and food. Nest quality was scored after 1, 3, 5, 9, and 24 h according to an established scale of 0–5, based on the degree of nestlet shredding, compaction, and organization^61^.

### Stone T-maze navigation

Water-motivated Stone T-maze navigation was assessed as a measure of learning, memory, and decision making, as well as physical function, as previously described^39^. The paradigm consists of a 2-day protocol, with training and a test day. On each day mice were transferred to the testing room for acclimation at least 30 min before the experiment. A metal reservoir, which was filled with room temperature water to a depth of ~2.2 cm (walkable depth, no swimming required), housed the ‘straight run’, comprised of an opaque entry box with a sliding door leading to an extended, straight clear acrylic alley chamber with a ceiling that ended in a dry, opaque goal box. Mice learn that moving through the straight clear chamber containing aversive water and exposure enables them to escape to the dry enclosed box. The mice were placed in the entry chamber and gently pushed into the straight alley with a plunger in the entry box, and the time required to enter the goal box was scored over six randomized, alternating trials. Successful completion of the straight run required mice to reach the goal box in 15 s or less, in 4 out of 6 trials. Mice unable to reach this criterion were excluded from further testing. The following day, a Stone T-Maze, consisting of an opaque entry box with a sliding door leading to a maze of clear acrylic alleys consisting of T-junctions, dead ends, and sliding guillotine doors (to prevent excessive backtracking), ending in a dry, opaque goal box, was placed in the metal reservoir containing 2.2 cm room temperature water. The mice were placed in the entry chamber and gently pushed into the maze with a plunger in the entry box, and the time required to enter the goal box and the number of errors committed while navigating the maze were scored over six randomized, alternating trials. An error was defined as the entry of the mouse’s entire body into an incorrect path, as delineated by the shoulder down. Successful completion of the maze run required mice to reach the goal box in 135 s or less, in 4 out of 6 trials. Mice unable to reach this criterion were excluded from further testing or analyses. For both the straight and maze runs, the first three randomized, alternating trials were followed by a 15-min break, concluding with the final three alternating trials. Between individual trials, mice were placed in a separate holding cage containing dry bedding material, a heating lamp, and a dry towel, followed by a return to their home cage.

### Statistical analyses

For NanoString data, each normalized gene was scaled by subtracting the mean and dividing by the standard deviation, and values were checked graphically for outliers and significant skewedness. The normalized and scaled NanoString data were analyzed using linear regression models and the false discovery rate (*q*-value) was used to adjust for multiple comparisons. DeepVenn/BioVenn was used to generate proportional Venn diagrams^63^. Ingenuity pathway analysis (IPA) was used to functionally annotate NanoString gene expression profiles. The Wilcoxon Rank Sum test was used for scRNA-seq differential gene expression analysis using the Seurat package. The Kolmogorov–Smirnov statistic was used for gene set enrichment analysis for the scRNA-seq data using GSEA software. Mass cytometry data were analyzed using generalized linear mixed models (GLMM) with multiple comparison testing. Behavioral data were analyzed using two-way ANOVA. For imaging and in vitro datasets, one- or two-group comparisons were analyzed using one- or two-sample tests, respectively. Associations between mass cytometry and behavioral data were interrogated using Spearman correlations. Graphpad Prism 9 and R were used for statistical analyses and graphing.

### Reporting summary

Further information on research design is available in the Nature Research Reporting Summary linked to this article.

## Supplementary information

Supplementary Information

Supplementary Dataset 1

Supplementary Dataset 2

Supplementary Dataset 3

Supplementary Movie 1

Reporting Summary

Description of Additional Supplementary Files

## Data Availability

scRNA-seq data generated in this study are deposited in Gene Expression Omnibus (GEO) under record GSE178957. An interactive website of the scRNA-seq data can be found at https://mayoxz.shinyapps.io/Brain/. Young and old mouse brain scRNA-seq data^36^ are publicly available at GEO under record GSE129788. Additional data and code included in this article will be provided by the corresponding author upon request. Source data are provided with this paper.

## Source data

Source data

## Code availability

As specified in the methods, the Seurat pipeline (v3.1.3)^35^ was used for scRNA-seq analyses. Markdown documents of the code used in this study will be provided by the corresponding author upon request.
