# Supplementary material for: Rejuvenation of the aged brain immune cell landscape in mice through p16-positive senescent cell clearance
Source: Nat Commun. 2022 Sep 27;13:5671. doi: 10.1038/s41467-022-33226-8 (PMC9515187; doi:10.1038/s41467-022-33226-8)
Supplement: Supplementary file 1 — Supplementary Information [file 41467_2022_33226_MOESM1_ESM.pdf]

Supplementary Information

Title: Rejuvenation of the aged brain immune cell landscape in mice through p16-positive senescent cell clearance

Authors and Affiliations:

1 Xu Zhang[1,2]
2 Vesselina M. Pearsall[1]
3 Chase M. Carver[3]
4 Elizabeth J. Atkinson[4]
5 Benjamin D. S. Clarkson[5]
6 Ethan M. Grund[6]
7 Michelle Baez-Faria[3,7]
8 Kevin D. Pavelko[8]
9 Jennifer M. Kachergus[9]
10 Thomas A. White[1]
11 Renee K. Johnson[5]
12 Courtney S. Malo[8]
13 Alan M. Gonzalez-Suarez[3]
14 Katayoun Ayasoufi[8]
15 Kurt O. Johnson[1]
16 Zachariah Tritz[8]
17 Cori E. Fain[8]
18 Roman H. Khadka[8]
19 Mikolaj Ogrodnik[1,10]
20 Diana Jurk[1,3]
21 Yi Zhu[1,3]
22 Tamara Tchkonia[1,3]
23 Alexander Revzin[3]
24 James L. Kirkland[1,3,11]
25 Aaron J. Johnson[8]
26 Charles L. Howe[5,12,13]
27 E. Aubrey Thompson[9]
28 Nathan K. LeBrasseur[1,2,3]
29 Marissa J. Schafer[1,3,5*]

[1]Robert and Arlene Kogod Center on Aging, Mayo Clinic, Rochester, MN, USA;
[2]Department of Physical Medicine and Rehabilitation, Mayo Clinic, Rochester, MN, USA;
[3]Department of Physiology and Biomedical Engineering, Mayo Clinic, Rochester, MN, USA;
[4]Division of Clinical Trials and Biostatistics, Department of Quantitative Health Sciences, Mayo Clinic, Rochester, MN, USA;
[5]Department of Neurology, Mayo Clinic, Rochester, MN, USA;
[6]Mayo Graduate School and Medical Scientist Training Program, Mayo Clinic, Rochester, MN, USA;
[7]Department of Laboratory Medicine and Pathology, Mayo Clinic, Rochester, MN, USA;
[8]Department of Immunology, Mayo Clinic, Rochester, MN, USA;
[9]Department of Cancer Biology, Mayo Clinic Comprehensive Cancer Center, Mayo Clinic, Jacksonville, FL, USA;
[10]Ludwig Boltzmann Research Group Senescence and Healing of Wounds, Vienna, Austria;
[11]Department of General Internal Medicine, Mayo Clinic, Rochester, MN, USA;
[12]Center for Multiple Sclerosis and Autoimmune Neurology, Mayo Clinic, Rochester, MN, USA
[13]Division of Experimental Neurology, Mayo Clinic, Rochester, MN, USA.
*Corresponding Author.

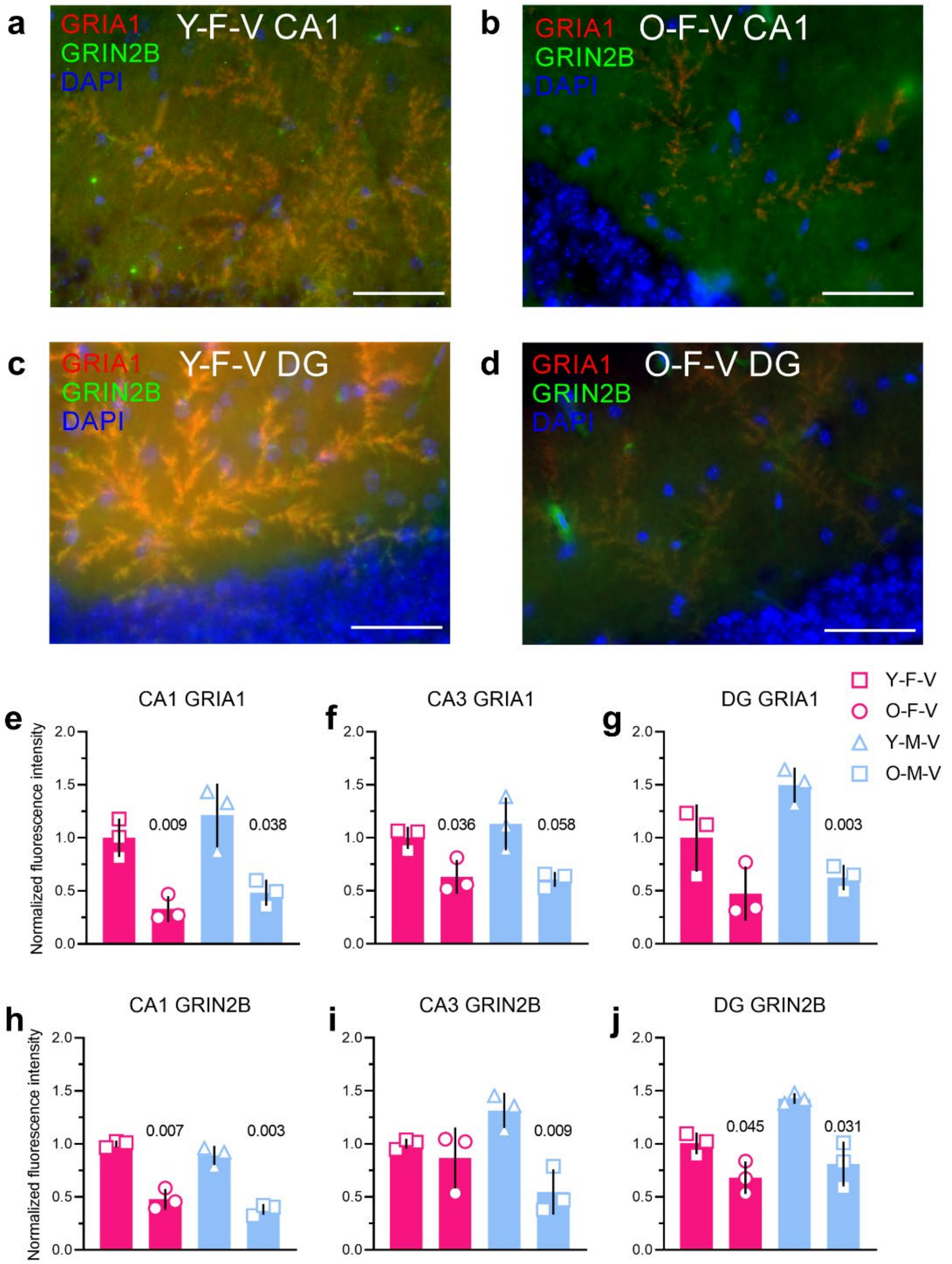

Supplementary Figure 1. **Confirmation of age-dependent downregulation of glutamatergic GRIA1 and GRIN2B receptor abundance across hippocampal subregions; related to figure 1.**

(a-d) Representative young *versus* old brain IF images of synaptic markers GRIA1 (red) and GRIN2B (green) in hippocampal sub-regions, including (a) young female CA1, (b) old female CA1, (c) young female dentate gyrus (DG), and (d) old female DG (20x objective; 50 µm scale bar). (e-g) Quantification of GRIA1-selective IF labeling intensity for (e) CA1, (f) CA3, and (g) DG hippocampal sub-regions in young and old mice. (h-j) Quantification of GRIN2B-selective IF labeling intensity for (h) CA1, (i) CA3, and (j) DG hippocampal sub-regions in young and old mice (n = 3 per group with 3-5 brain sections quantified per animal in one experiment; Y-F-V = six months, female, vehicle; O-F-V = 24 months, female, vehicle; Y-M-V = six months, male, vehicle; O-M-V = 24 months, male, vehicle; mean ± SEM; two-tailed unpaired t-tests, female and male samples analyzed separately).

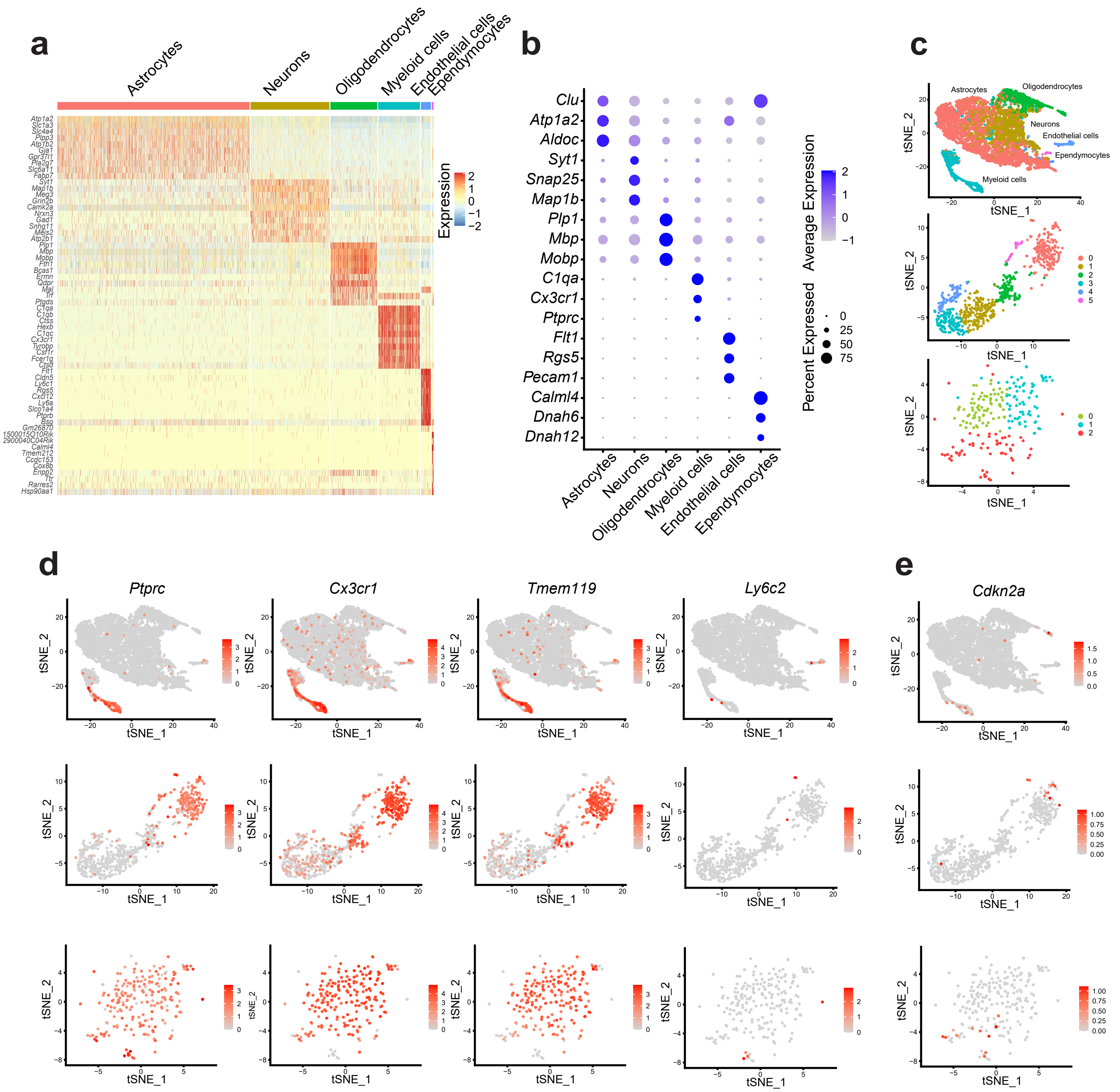

a
Astrocytes
Neurons
Oligodendrocytes
Myeloid cells
Endothelial cells
Ependymocytes
Atp1a2
Slc1a3
Slc4a4
Plpp3
Atp1b2
Gja1
Gpr37l1
Pla2g7
Slc6a11
Fabp7
Syt1
Map1b
Meg3
Grin2b
Camk2a
Nrxn3
Gad1
Snhg11
Meis2
Atp2b1
Plp1
Mbp
Mobp
Fth1
Bcas1
Ermn
Qdpr
Mal
Trf
Ptgds
C1qa
C1qb
Ctss
Hexb
C1qc
Cx3cr1
Tyrobp
Csf1r
Fcer1g
Ctsd
Flt1
Cldn5
Ly6c1
Rgs5
Cxcl12
Ly6a
Slco1a4
Ptprb
Bsg
Gm26870
1500015O10Rik
2900040C04Rik
Calml4
Tmem212
Ccdc153
Cox8b
Enpp2
Ttr
Rarres2
Hsp90aa1
Expression
b
Clu
Atp1a2
Aldoc
Syt1
Snap25
Map1b
Plp1
Mbp
Mobp
C1qa
Cx3cr1
Ptprc
Flt1
Rgs5
Pecam1
Calml4
Dnah6
Dnah12
Astrocytes
Neurons
Oligodendrocytes
Myeloid cells
Endothelial cells
Ependymocytes
Average Expression
Percent Expressed
c
Astrocytes
Oligodendrocytes
Neurons
Endothelial cells
Ependymocytes
Myeloid cells
tSNE_1
tSNE_2
d
Ptprc
Cx3cr1
Tmem119
Ly6c2
e
Cdkn2a
tSNE_1
tSNE_2

Supplementary Figure 2. **Single-cell RNA-sequencing reveals age-related *p16*-positive senescent brain myeloid cell population; related to figure 2.**

(a) Heatmap of cluster-specific genes for the six clusters identified in mouse brain. (b) Dot plot of canonical cell type markers for the six clusters. Dot size represents the percentage of cells that express the marker, and the color represents the expression level of the marker. (c) Cell population selection process based on *Cdkn2a/p16*^{Ink4a} expression (reference panel e). TOP: tSNE plot of cell types identified by scRNA-seq in mouse brain. (e) TOP: Myeloid cells contain the greatest frequency of *Cdkn2a*-positive cells. (c) MIDDLE: tSNE plot of the myeloid cell compartment, revealing six subclusters. (e) MIDDLE: Myeloid subcluster zero contained the greatest frequency of *Cdkn2a*-positive cells. (c) BOTTOM: tSNE plot of myeloid subcluster zero, revealing three subclusters. (e) BOTTOM: Myeloid subcluster zero contained the greatest frequency of *Cdkn2a/p16*-positive cells. (d) Expression of immune (*Ptprc),* myeloid *(Cx3cr1*), microglial (*Tmem119*), monocyte (*Ly6c2*)*,* (e) and senescence (*Cdkn2a/p16*^{Ink4a}*)* markers across the sequential clustering schema*.* Each dot represents an individual cell, and the color scale bar represents the expression level of each gene.

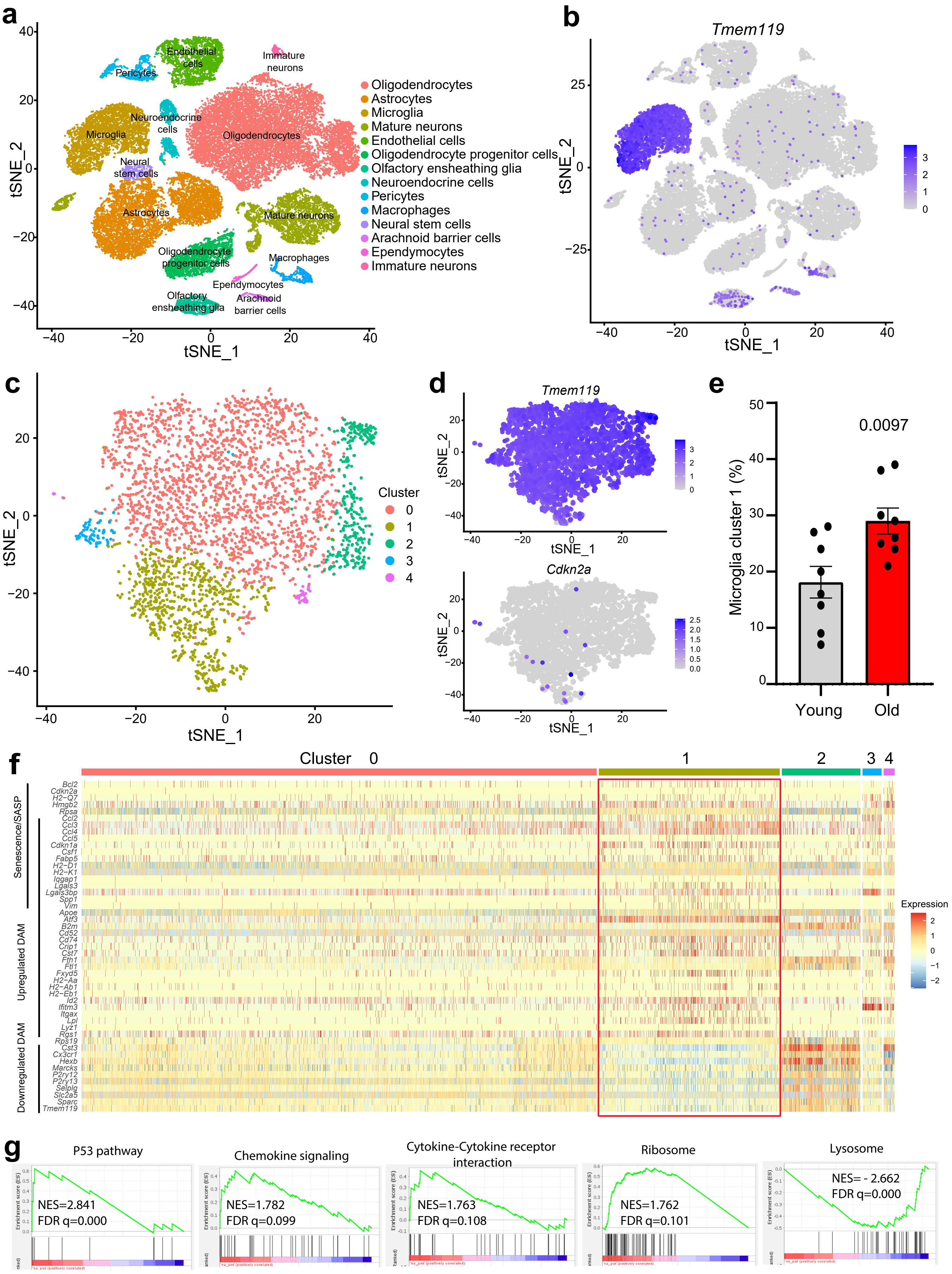
a
Endothelial cells
Immature neurons
Pericytes
Neuroendocrine cells
Microglia
Oligodendrocytes
Neural stem cells
Astrocytes
Mature neurons
Oligodendrocyte progenitor cells
Macrophages
Ependymocytes
Olfactory ensheathing glia
Arachnoid barrier cells
Oligodendrocytes
Astrocytes
Microglia
Mature neurons
Endothelial cells
Oligodendrocyte progenitor cells
Olfactory ensheathing glia
Neuroendocrine cells
Pericytes
Macrophages
Neural stem cells
Arachnoid barrier cells
Ependymocytes
Immature neurons
tSNE_1
tSNE_2
b
Tmem119
c
Cluster
d
Tmem119
Cdkn2a
e
0.0097
Microglia cluster 1 (%)
Young
Old
f
Cluster 0 1 2 3 4
Senescence/SASP
Upregulated DAM
Downregulated DAM
Bcl2
Cdkn2a
H2−Q7
Hmgb2
Rpsa
Ccl2
Ccl3
Ccl4
Ccl5
Cdkn1a
Csf1
Fabp5
H2−D1
H2−K1
Iqgap1
Lgals3
Lgals3bp
Spp1
Vim
Apoe
Atf3
B2m
Cd52
Cd74
Crip1
Cst7
Fth1
Ftl1
Fxyd5
H2−Aa
H2−Ab1
H2−Eb1
Id2
Ifitm3
Itgax
Lpl
Lyz1
Rgs1
Rps19
Cst3
Cx3cr1
Hexb
Marcks
P2ry12
P2ry13
Selplg
Slc2a5
Sparc
Tmem119
Expression
g
P53 pathway
NES=2.841
FDR q=0.000
Chemokine signaling
NES=1.782
FDR q=0.099
Cytokine-Cytokine receptor interaction
NES=1.763
FDR q=0.108
Ribosome
NES=1.762
FDR q=0.101
Lysosome
NES= - 2.662
FDR q=0.000

Supplementary Figure 3. **Analysis of Ximerakis *et. al.* single-cell RNA-sequence data confirms the identity of a senescent microglia population that increases in the aged mouse brain.** (a) T-distributed stochastic neighbor embedding (tSNE) plot of cell types identified by scRNA-seq in young (2-3 months) and old (21-22 months) mouse brain samples (n = 8 per age group). (b) tSNE plot demonstrating *Tmem119*-positive microglial population that was applied to unbiased subclustering, (c) which revealed five microglia clusters. (d) tSNE plot showing *Tmem119 and Cdkn2a/p16*^{Ink4a} expression in the five microglial clusters. Each dot represents an individual cell, and the clusters match panel C. The color scale bar represents the expression level. (e) Young versus old cell abundance of microglial cluster one, which contained the greatest frequency of *Cdkn2a*-positive microglia (n = 8 per age group; mean $\pm$ SEM; two-tailed unpaired t-test). (f) Heatmap of expression levels of senescence, SASP, and disease-associated (DAM) genes in the five microglia clusters. The molecular profile of Ximerakis's microglial subcluster one demonstrates remarkable similarity to the molecular profile of senescent myeloid cluster two depicted in Figure 2. (g) Pathways enriched in microglia cluster one identified by GSEA with the normalized enrichment score (NES) and FDR q-values indicated in each panel.

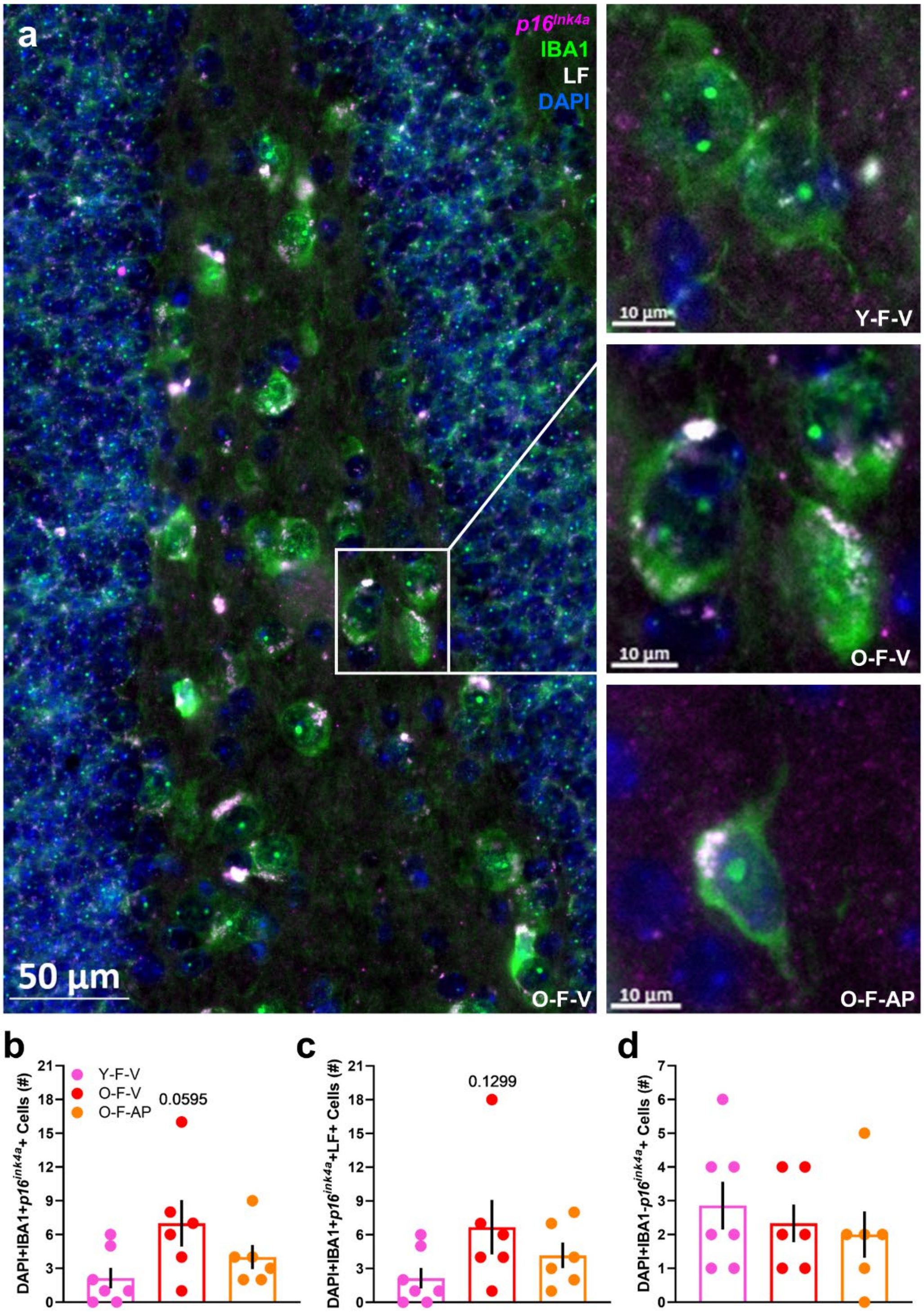

Supplementary Figure 4. ***p16*-positive myeloid cells exhibiting lysosomal stress accumulate in the aged dentate gyrus.** (a) RNAish, immunofluorescence, autofluorescence, and DAPI were used to detect $p16^{Ink4a}$, IBA1, lipofuscin (LF) granules, and nuclei, respectively, in the hippocampal dentate gyrus hilus of 6-month-old female brain sections treated with vehicle (Y-F-V), 24-month-old female brain sections treated with vehicle (O-F-V), and 24-month-old female brain sections treated with AP20187 (O-F-AP). Images are representative of n = 6-7 per condition. Image quantification demonstrating counts of hilar (b) $p16^{Ink4a}$+ myeloid cells (DAPI+IBA1+$p16^{Ink4a}$+), (c) $p16^{Ink4a}$+ myeloid cells harboring lysosomal stress granules (DAPI+IBA1+$p16^{Ink4a}$+LF+), and (d) non-myeloid $p16^{Ink4a}$+ cells (DAPI+IBA1-$p16^{Ink4a}$+) (n = 7 Y-F-V, n = 6 O-F-V, n = 6 O-F-AP; mean ± SEM; one-way ANOVA with multiple comparison testing).

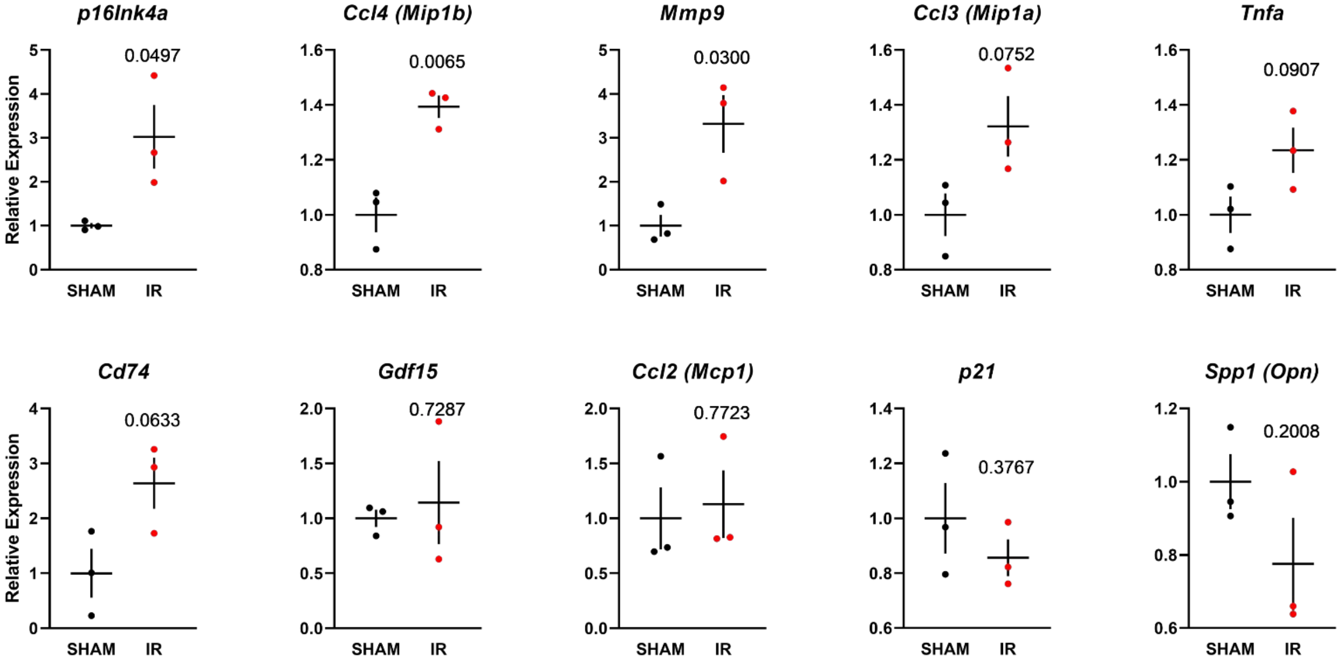

Supplementary Figure 5. **Confirmation of a senescent gene expression signature in primary brain myeloid cells exposed to irradiation; related to figure 3.** RT-PCR analysis of senescence and SASP factors in CD45+CD11b+ brain myeloid cells two weeks post irradiation exposure or sham conditions. Target gene expression was normalized to *Hprt* and the average target gene expression of the Sham condition (n = 3 sham cultures, n = 3 irradiated cultures; mean + SEM; two-tailed unpaired t-tests).

Supplementary Figure 6. **Peripheral immune cell migration towards SASP-containing conditioned media from senescent brain myeloid cells; related to figure 3.** See separate video file and legend.

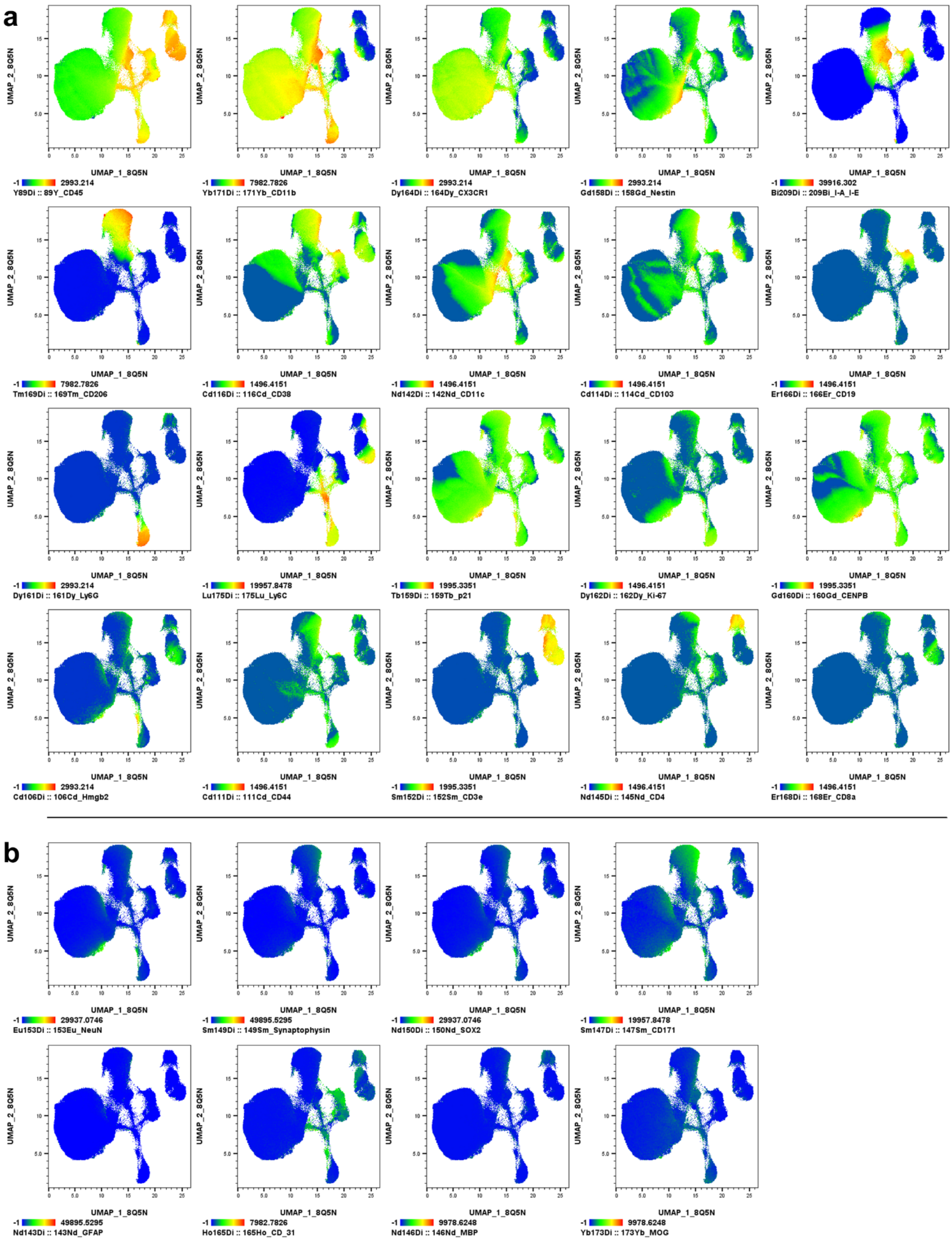

Supplementary Figure 7. **Mass cytometry makers defining the CD45+ brain cell landscape; related to figure 4.** Overlayed on the UMAP described in figure 4 are single marker presentations of the (a) twenty immune markers and (b) lack of or low-level signal for eight markers utilized for negative selection in data preprocessing.

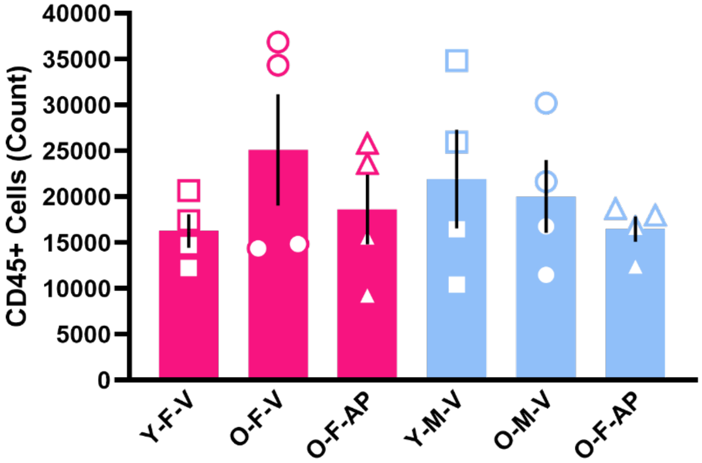

Supplementary Figure 8. **Total counts of CD45+ cells analyzed by mass cytometry per age and treatment group; related to figure 4.** Total counts of cells applied to UMAP dimensionality reduction and FlowSOM-guided clustering in figure 4. (n = 4; Y-F-V = six months, female, vehicle; O-F-V = 24 months, female, vehicle; O-F-AP = 24 months, female, AP20187; Y-M-V = six months, male, vehicle; O-M-V = 24 months, male, vehicle; O-M-AP = 24 months, male, AP20187; mean + SEM; one-way ANOVA; female and male samples analyzed separately).

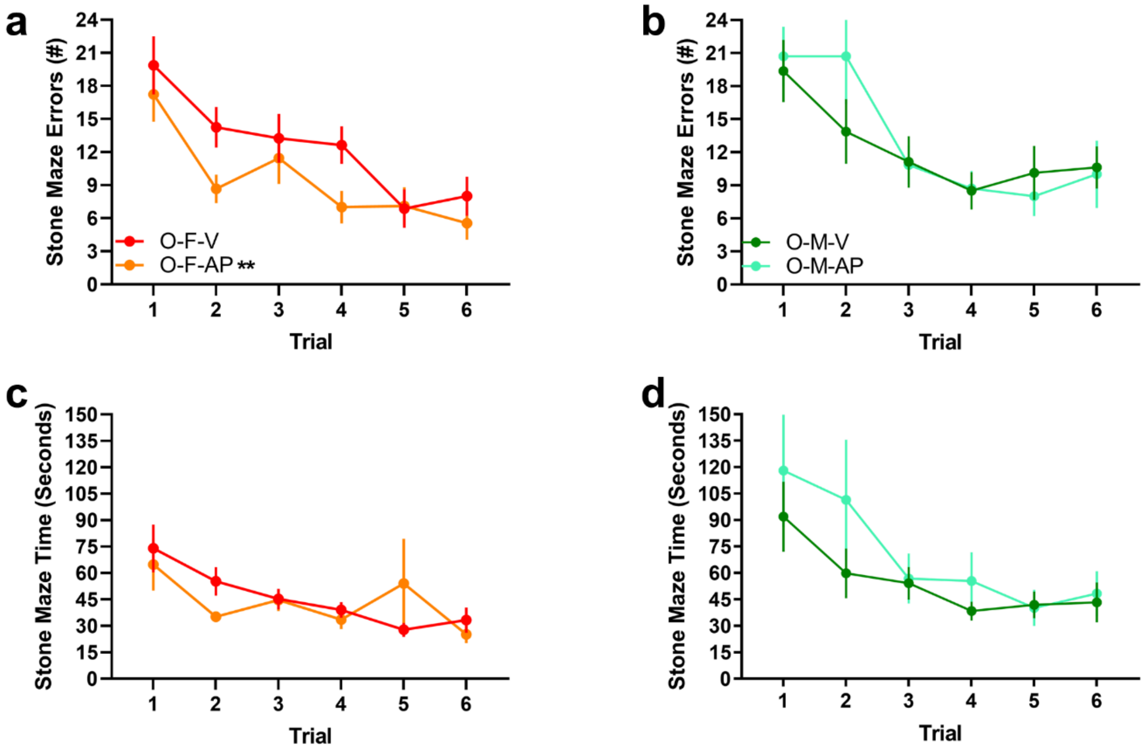

Supplementary Figure 9. **Stone maze navigation is improved in aged females following systemic targeting of p16-positive senescent cells.**

(a) The water-motivated stone maze task was used to probe hippocampal-related decision making, learning and memory, and physical function. The number of (a-b) errors and (c-d) time (seconds) required to complete the maze were scored among 24-month-old (O) (a, c) females and (b, d) males treated with vehicle (V) or AP20187 (AP) (n = 8 Y-F-V, n = 8 O-F-V, n = 9 O-F-AP, n = 7 Y-M-V, n = 8 O-M-V, n = 7 O-M-AP; mean ± SEM; two-way ANOVA; **p = 0.009).
