# Supplementary material for: Rejuvenation of the aged brain immune cell landscape in mice through p16-positive senescent cell clearance
Source: Nat Commun. 2022 Sep 27;13:5671. doi: 10.1038/s41467-022-33226-8 (PMC9515187; doi:10.1038/s41467-022-33226-8)
Supplement: Supplementary file 6 — Reporting Summary [file 41467_2022_33226_MOESM6_ESM.pdf]

nature portfolio

Corresponding author(s): Marissa Schafer
Last updated by author(s): 23 Aug. 2022

Reporting Summary

Nature Portfolio wishes to improve the reproducibility of the work that we publish. This form provides structure for consistency and transparency in reporting. For further information on Nature Portfolio policies, see our Editorial Policies and the Editorial Policy Checklist.

Statistics

For all statistical analyses, confirm that the following items are present in the figure legend, table legend, main text, or Methods section.

n/a	Confirmed	
☐	☒	The exact sample size (*n*) for each experimental group/condition, given as a discrete number and unit of measurement
☐	☒	A statement on whether measurements were taken from distinct samples or whether the same sample was measured repeatedly
☐	☒	The statistical test(s) used AND whether they are one- or two-sided<br>*Only common tests should be described solely by name; describe more complex techniques in the Methods section.*
☒	☐	A description of all covariates tested
☐	☒	A description of any assumptions or corrections, such as tests of normality and adjustment for multiple comparisons
☐	☒	A full description of the statistical parameters including central tendency (e.g. means) or other basic estimates (e.g. regression coefficient) AND variation (e.g. standard deviation) or associated estimates of uncertainty (e.g. confidence intervals)
☐	☒	For null hypothesis testing, the test statistic (e.g. *F*, *t*, *r*) with confidence intervals, effect sizes, degrees of freedom and *P* value noted<br>*Give P values as exact values whenever suitable.*
☒	☐	For Bayesian analysis, information on the choice of priors and Markov chain Monte Carlo settings
☒	☐	For hierarchical and complex designs, identification of the appropriate level for tests and full reporting of outcomes
☒	☐	Estimates of effect sizes (e.g. Cohen's *d*, Pearson's *r*), indicating how they were calculated

Our web collection on statistics for biologists contains articles on many of the points above.

Software and code

Policy information about availability of computer code

Data collection	Excel (Microsoft 365 for enterprise Version 16.0.15330.20196) was used for data collection.
Data analysis	FlowJo v10.8.1, R v4.1.2, R v4.0.3, R v3.6.0, HiSeq Control Software (HCS) 3.3.52, Cell Ranger v3.0, Seurat v3.1.3, Seurat v4.0.2, GSEA version 4.0.3, ImageJ v1.53, and ShinyCell v2.1.0 were used for data analysis.<br><br>R Markdown documents containing analysis codes are provided as supplementary files "R Markdown for Zhang scRNAseq analysis.html" and "R Markdown for Ximerakis scRNAseq analysis.html".

For manuscripts utilizing custom algorithms or software that are central to the research but not yet described in published literature, software must be made available to editors and reviewers. We strongly encourage code deposition in a community repository (e.g. GitHub). See the Nature Portfolio guidelines for submitting code & software for further information.

Data

Policy information about availability of data

All manuscripts must include a data availability statement. This statement should provide the following information, where applicable:

- Accession codes, unique identifiers, or web links for publicly available datasets
- A description of any restrictions on data availability
- For clinical datasets or third party data, please ensure that the statement adheres to our policy

scRNA-seq data used in this study are deposited in Gene Expression Omnibus (GEO) under record GSE178957. (Reviewer's key to access prior to publication: ypmfmwmajlglrah). An interactive website of the scRNA-seq data can be found at https://mayoxz.shinyapps.io/Brain/. Ximerakis et. al. young and old mouse brain scRNA-seq dataset is publicly available at GEO under record GSE129788. As discussed with reviewers, we are not aware of a publicly available database that hosts

mass cytometry data. Summary data are provided, and additional information can be provided upon reasonable request.

Field-specific reporting

Please select the one below that is the best fit for your research. If you are not sure, read the appropriate sections before making your selection.

- [x] Life sciences
- [ ] Behavioural & social sciences
- [ ] Ecological, evolutionary & environmental sciences

For a reference copy of the document with all sections, see nature.com/documents/nr-reporting-summary-flat.pdf

Life sciences study design

All studies must disclose on these points even when the disclosure is negative.

Sample size	Sample sizes were determined according to current standards used for mice in experimental biology, based on the minimal amount of mice required to detect significance with an alpha set at 0.05 in a standard powered experiment (PMID: 28230051).
Data exclusions	Samples in which severe splenomegaly were observed at the time of necropsy were excluded from cytometry experiments.
Replication	Biological replicates are described in the figure legends.
Randomization	Mice were randomized to treatment groups based on body weight and/or body composition.
Blinding	Experimenters were blinded to group allocations during experiments/outcome assessment.

Reporting for specific materials, systems and methods

We require information from authors about some types of materials, experimental systems and methods used in many studies. Here, indicate whether each material, system or method listed is relevant to your study. If you are not sure if a list item applies to your research, read the appropriate section before selecting a response.

Materials & experimental systems

n/a	Involved in the study	
[ ]	[x]	Antibodies
[x]	[ ]	Eukaryotic cell lines
[x]	[ ]	Palaeontology and archaeology
[ ]	[x]	Animals and other organisms
[x]	[ ]	Human research participants
[x]	[ ]	Clinical data
[x]	[ ]	Dual use research of concern

Methods

n/a	Involved in the study	
[x]	[ ]	ChIP-seq
[x]	[ ]	Flow cytometry
[x]	[ ]	MRI-based neuroimaging

Antibodies

Antibodies used

IF: anti-IBA (Wako, Catalog# 019-19741, 1:100)
IF: Alexa Fluor 488 goat anti-rabbit IgG (H+L) (ThermoFisher/Invitrogen, Catalog# A11008, 1:150)
IF: anti-glutamate receptor 1 subtype (Abcam, Catalog# ab183797, 1:1000)
IF: anti-NMDAR2B (Abcam, Catalog# ab93610, 1:500)
IF: AlexaFluor488 donkey anti-mouse IgG (Jackson Immunoresearch, Catalog# 715-546-151, 1:250)
IF: Rhodamine Red-X donkey anti-rabbit IgG (Jackson Immunoresearch, Catalog# 711-296-15, 1:250)
Mass Cytometry: anti-CD103 (Abcam, Catalog# ab25198, 1:100)
Mass Cytometry: anti-CD11b (Biolegend, Catalog# 101249, 1:400)
Mass Cytometry: anti-CD11c (Fluidigm, Catalog# 3142003B, 1:100)
Mass Cytometry: anti-CD171 (Abcam, Catalog# ab24345, 1:200)
Mass Cytometry: anti-CD19 (Fluidigm, Catalog# 3166015B, 1:200)
Mass Cytometry: anti-CD206 (Fluidigm, Catalog# 3169021B, 1:100)
Mass Cytometry: anti-CD31 (Fluidigm, Catalog# 3165013B, 1:100)
Mass Cytometry: anti-CD38 (Biolegend, Catalog# 102702, 1:100)
Mass Cytometry: anti-CD3e (Fluidigm, Catalog# 3152004B, 1:400)
Mass Cytometry: anti-CD4 (Fluidigm, Catalog# 3145002B, 1:200)
Mass Cytometry: anti-CD44 (Biolegend, Catalog# 103051, 1:100)
Mass Cytometry: anti-CD45 (Fluidigm, Catalog# 3089005B, 1:200)
Mass Cytometry: anti-CD8a (Fluidigm, Catalog# 3168003B, 1:400)
Mass Cytometry: anti-CENPB (Abcam, Catalog# ab25734, 1:100)
Mass Cytometry: anti-CX3CR1 (Fluidigm, Catalog# 3164023B, 1:200)

Mass Cytometry: anti-GFAP (Fluidigm, Catalog# 3143022B, 1:200)
Mass Cytometry: anti-Hmgb2 (Abcam, Catalog# ab248543, 1:100)
Mass Cytometry: anti-I-A/I-E (Fluidigm, Catalog# 3209006B, 1:200)
Mass Cytometry: anti-Ki-67 (Fluidigm, Catalog# 3162012B, 1:100)
Mass Cytometry: anti-Ly6C (Biolegend, Catalog# 128039, 1:400)
Mass Cytometry: anti-Ly6G (Biolegend, Catalog# 127637, 1:400)
Mass Cytometry: anti-MBP (Abcam, Catalog# ab62631, 1:100)
Mass Cytometry: anti-MOG (ThermoFisher, Catalog# PA5-95602, 1:100)
Mass Cytometry: anti-Nestin (Abcam, Catalog# ab6142, 1:100)
Mass Cytometry: anti-NeuN (Abcam, Catalog# ab177487, 1:80)
Mass Cytometry: anti-p21 (Santa Cruz, Catalog# sc-6246, 1:100)
Mass Cytometry: anti-SOX2 (Sigma-Aldrich, Catalog# S9072, 1:100)
Mass Cytometry: anti-SYP (Abcam, Catalog# EP1098Y, 1:200)

Validation

Immunofluorescence Imaging
anti-IBA1 (Wako, 1:100)
Species: Rabbit
Reactivity: Human, Mouse, Rat
Applications: ICC, IHC

Immunofluorescence Imaging
anti-rabbit IgG (H+L)-Alexa Fluor 488 (ThermoFisher, 1:150)
Species: Goat
Reactivity: Rabbit
Applications: ICC, IHC, Flow Cytometry

Immunofluorescence Imaging
anti-glutamate receptor 1 subtype (AMPA) (Abcam, 1:1000)
Species: Rabbit
Reactivity: Mouse, Rat, Human
Applications: ICC, IHC

Immunofluorescence Imaging
anti-NMDAR2B (Abcam, 1:500)
Species: Mouse
Reactivity: Mouse, Rat, Human
Applications: ICC, IHC

Immunofluorescence Imaging
anti-mouse IgG F(ab')2 fragment, AlexaFluor488 conjugated (Jackson Immunoresearch, 1:250)
Species: Donkey
Reactivity: Mouse
Applications: ICC, IHC

Immunofluorescence Imaging
anti-rabbit IgG F(ab')2 fragment, Rhodamine Red-X conjugated (Jackson Immunoresearch, 1:250)
Species: Donkey
Reactivity: Rabbit
Applications: ICC, IHC

Mass Cytometry
anti-CD45-089Y (Fluidigm, clone 30-F11, 1:200)
Species: Rat
Reactivity: Mouse
Applications: Mass Cytometry

Mass Cytometry
anti-Hmgb2-106Cd (Abcam, clone EPR6302, 1:100)
Species: Rabbit
Reactivity: Mouse, Rat, Human
Applications: IHC-P, WB

Mass Cytometry
anti-CD44-111Cd (Biolegend, clone IM7, 1:100)
Species: Rat
Reactivity: Mouse, Human, Chimpanzee, Baboon, Cynomolgus, Rhesus, Squirrel Monkey, Horse, Cattle, Swine, Dog, Cat

Applications: Mass or Flow Cytometry

Mass Cytometry
anti-CD103-114Cd (Abcam, clone 2E7, 1:100)
Species: Armenian Hamster
Reactivity: Mouse
Applications: Mass or Flow Cytometry

Mass Cytometry
anti-CD38-116Cd (Biolegend, clone 90, 1:100)
Species: Rat
Reactivity: Mouse
Applications: Mass or Flow Cytometry, IHC-F

Mass Cytometry
anti-CD11c-142Nd (Fluidigm, clone N418, 1:100)
Species: Armenian Hamster
Reactivity: Mouse
Applications: Mass Cytometry

Mass Cytometry
anti-GFAP-143Nd (Fluidigm, clone GA5, 1:200)
Species: Mouse
Reactivity: Cross
Applications: Mass Cytometry

Mass Cytometry
anti-CD4-145Nd (Fluidigm, clone RM4-5, 1:200)
Species: Rat
Reactivity: Mouse
Applications: Mass Cytometry

Mass Cytometry
anti-CD171-147Sm (Abcam, clone 2C2, 1:200)
Species: Mouse
Reactivity: Mouse, Rat, Human
Applications: ICC/IF, IHC-P, WB

Mass Cytometry
anti-SYP-149Sm (Abcam, clone EP1098Y, 1:200)
Species: Rabbit
Reactivity: Mouse, Rat, Human
Applications: Flow Cytometry, WB, ICC/IF, IHC-P

Mass Cytometry
anti-CD3e-152Sm (Fluidigm, clone 145-2C11, 1:400)
Species: Armenian Hamster
Reactivity: Mouse
Applications: Mass Cytometry

Mass Cytometry
anti-Nestin-158Gd (Abcam, clone Rat-401, 1:100)
Species: Mouse
Reactivity: Mouse, Rat, Human
Applications: IHC-P, WB

Mass Cytometry
anti-p21-159Tb (Santa Cruz, clone F-5, 1:100)
Species: Mouse
Reactivity: Mouse
Applications: Flow Cytometry, WB, ICC/IF

Mass Cytometry
anti-CENPB-160Gd (Abcam, clone ab25734, 1:100)
Species: Rabbit
Reactivity: Human, Mouse, Rat, Sheep, Hamster
Applications: WB, ICC/IF

Mass Cytometry
anti-Ly6G-161Dy (Biolegend, clone 1A8, 1:400)
Species: Rat
Reactivity: Mouse
Applications: Mass or Flow Cytometry

Mass Cytometry
anti-Ki-67-162Dy (Fluidigm, clone B56, 1:100)
Species: Mouse
Reactivity: Rat, Mouse, Human, Porcine
Applications: Mass Cytometry

Mass Cytometry
anti-CX3CR1-164Dy (Fluidigm, clone SA011F11, 1:200)
Species: Mouse
Reactivity: Mouse
Applications: Mass Cytometry

Mass Cytometry
anti-CD31-165Ho (Fluidigm, clone 390, 1:100)
Species: Rat
Reactivity: Mouse
Applications: Mass Cytometry

Mass Cytometry
anti-CD19-166Er (Fluidigm, clone 6D5, 1:200)
Species: Rat
Reactivity: Mouse
Applications: Mass Cytometry

Mass Cytometry
anti-CD8a-168Er (Fluidigm, clone 53-6.7, 1:400)
Species: Rat
Reactivity: Mouse
Applications: Mass Cytometry

Mass Cytometry
anti-CD206-169Tm (Fluidigm, clone C068C2, 1:100)
Species: Rat
Reactivity: Mouse
Applications: Mass Cytometry

Mass Cytometry
anti-CD11b-171Yb (Biolegend, clone M1/70, 1:400)
Species: Rat
Reactivity: Mouse, Human, Chimpanzee, Baboon, Cynomolgus, Rhesus, Rabbit
Applications: Mass or Flow Cytometry

Mass Cytometry
anti-Ly6C-175Lu (Biolegend, clone HK1.4, 1:400)
Species: Rat
Reactivity: Mouse
Applications: Mass or Flow Cytometry

Mass Cytometry
anti-I-A/I-E-209Bi (Fluidigm, clone M5/114.15.2, 1:200)
Species: Rat
Reactivity: Mouse
Applications: Mass Cytometry

Mass Cytometry
anti-MBP-146Nd (Abcam, clone MBP101, 1:100)
Species: Human
Reactivity: Mouse, Rat, Human
Applications: ICC, Flow Cytometry

Mass Cytometry
anti-MOG-173Yb (ThermoFisher, PA5-95602, 1:100)

Species: Rabbit
Reactivity: Mouse, Rat, Human
Applications: IHC

Mass Cytometry
anti-SOX2-150Nd (Sigma-Aldrich, S9072, 1:100)
Species: Rabbit
Reactivity: Mouse, Rat, Human
Applications: IHC, WB

Mass Cytometry
anti-NeuN-153Eu (Abcam, clone EPR12763, 1:80)
Species: Rabbit
Reactivity: Mouse, Rat, Sheep, Goat, Cat, Dog, Human, Zebrafish, Common marmoset
Applications: Flow Cytometry, IHC, WB, ICC

Animals and other organisms

Policy information about studies involving animals; ARRIVE guidelines recommended for reporting animal research

Laboratory animals	Female and male C57BL/6 and heterozygous p16-InkAttac mice maintained on C57BL/6 background were used for these studies. Mice were 6- or 24-months-old.<br>Splenocytes from 1-2 month old female and male C57BL/6-Tg(CAG-EGFP)131Osb/LeySopJ mice were used for in vitro studies.<br>Mice were group-housed in ventilated cages with a constant temperature of 25C, 30-70% humidity, a 12-hour light/dark cycle, and provided standard chow.
Wild animals	No wild animals were used.
Field-collected samples	No field collected animals were used.
Ethics oversight	Mouse experiments were performed under protocols approved by Mayo Clinic Institutional Animal Care and Use Committee.

Note that full information on the approval of the study protocol must also be provided in the manuscript.
