# Supplementary material for: Rejuvenation of the aged brain immune cell landscape in mice through p16-positive senescent cell clearance
Source: Nat Commun. 2022 Sep 27;13:5671. doi: 10.1038/s41467-022-33226-8 (PMC9515187; doi:10.1038/s41467-022-33226-8)
Supplement: Supplementary file 7 — Description of Additional Supplementary Files [file 41467_2022_33226_MOESM7_ESM.pdf]

**Description of Additional Supplementary Files**

File Name: Supplementary Dataset 1

Description: NanoString Panel (excel file). nCounter experimental and control genes analyzed in this study.

File Name: Supplementary Dataset 2

Description: RT-PCR primers used in Supplementary Figure 5 (excel file). Panel of senescence -related and housekeeping RT-PCR primers used in this study.

File Name: Supplementary Dataset 3

Description: Mass Cytometry Panel (excel file). Mass cytometry panel information, including label, scale, and identity information for each marker used in this study.

File Name: Supplementary Movie 1

Description: (Figure 6.) Peripheral immune cell migration towards SASP-containing conditioned media from senescent brain myeloid cells; related to figure 3. Representative video of GFP+ splenocyte migration toward conditioned media from senescent brain myeloid cells (right side) versus conditioned media from control brain myeloid cells (left side) in a microfluidic migration chamber using an IncuCyte system for individual cell tracing over a 12-hour period.
